# Inflammation, coagulation, and nutritional reserves refine 90-day prognostic stratification after ruptured intracranial aneurysm repair

**DOI:** 10.3389/fnut.2026.1918951

**Published:** 2026-09-16

**Authors:** Jian Li, Zhen Wang, Hongwen Li, Xinyu Yang

**Affiliations:** 1 Department of Neurosurgery, Tianjin Medical University General Hospital, Heping, Tianjin, China; 2 Department of Neurosurgery, Tianjin Huanhu Hospital, Jinnan, Tianjin, China

**Keywords:** aneurysmal subarachnoid hemorrhage, d-dimer, neurological recovery, prognostic nutritional index, retrospective study, ruptured intracranial aneurysm, systemic immune-inflammation index

## Abstract

**Background:**

Ruptured intracranial aneurysm is a major cause of neurological disability. Existing prognostic models primarily rely on neurological and radiological severity and may overlook systemic biological alterations.

**Objective:**

To evaluate whether integrating systemic immune-inflammation index (SII), D-dimer, and prognostic nutritional index (PNI) with clinical-radiological factors improves prediction of 90-day unfavorable neurological recovery after aneurysm repair.

**Methods:**

This single-center retrospective cohort study included 209 patients with aneurysmal subarachnoid hemorrhage undergoing clipping or coiling between January 2023 and December 2025. The primary outcome was unfavorable 90-day functional recovery (modified Rankin Scale 3–6). Admission biomarkers were analyzed using multivariable logistic regression. An admission model and an updated in-hospital model incorporating delayed cerebral ischemia (DCI) were developed and evaluated using discrimination, calibration, decision curve analysis, and bootstrap validation.

**Results:**

Among 209 patients, 66 (31.6%) had unfavorable outcomes. Independent predictors included Hunt–Hess grade IV–V (aOR 3.85, 95% CI 1.84–8.06), modified Fisher grade 3–4 (aOR 2.76, 95% CI 1.28–5.95), acute hydrocephalus (aOR 2.62, 95% CI 1.25–5.48), higher SII (aOR 1.32 per 500-unit increase), higher D-dimer (aOR 1.22 per 1 mg/L increase), and lower PNI (aOR 0.68 per 5-point increase). The admission model achieved an AUC of 0.82 (95% CI 0.76–0.88), while the updated model incorporating DCI improved the AUC to 0.86 (95% CI 0.80–0.91), with an optimism-corrected AUC of 0.83 after bootstrap validation.

**Conclusion:**

An integrated model combining inflammatory, coagulation, nutritional, and clinical-radiological factors improved risk stratification after ruptured intracranial aneurysm repair. Incorporation of DCI provided additional prognostic value, although external validation is needed.

## 1 Introduction

Aneurysmal subarachnoid hemorrhage (aSAH) remains a devastating cerebrovascular emergency, with substantial mortality and long-term disability that disproportionately affect working-age populations (1). Contemporary guidelines emphasize that comprehensive management targeting early brain injury, delayed cerebral ischemia (DCI), and systemic complications is essential to optimize functional recovery (2, 3). Despite advances in surgical clipping, endovascular coiling, and neurocritical care, many patients experience persistent neurological deficits even after successful aneurysm occlusion (4). Accurate early identification of high-risk patients therefore remains an urgent priority to guide perioperative monitoring and individualized interventions.

Current prognostic tools such as the Hunt–Hess, World Federation of Neurosurgical Societies (WFNS), and modified Fisher grades are widely applied in clinical practice (5). These scores primarily capture initial neurological impairment and radiological hemorrhage extent, providing a static picture that does not reflect the dynamic, multisystem pathophysiological cascades triggered by aneurysm rupture (5, 6). Functional recovery is influenced not only by the primary bleeding event but also by secondary insults including early brain injury, neuroinflammation, microthrombus formation, and postictal hypercatabolism, all of which critically affect tissue repair and neurological outcomes (6, 7). Because, conventional grading systems omit systemic biochemical responses, their prognostic capacity remains inherently limited.

Accumulating evidence suggests that systemic biological markers reflecting inflammatory activation, coagulation abnormalities, and host nutritional reserve offer valuable prognostic information (8, 9). The systemic immune-inflammation index (SII) integrates neutrophils, platelets, and lymphocytes, thereby capturing the interplay between hyperinflammation and immune suppression (10–12). D-dimer reflects prothrombotic tendency and microthrombus activation, mechanisms closely implicated in DCI and microcirculatory disturbance (13, 14). The prognostic nutritional index (PNI) represents systemic metabolic reserve and immune resilience, serving as a marker of host recovery capacity (15, 16). Although individual markers within these pathways have been separately associated with poor outcomes, most prior investigations focused on isolated biological dimensions. Whether integrating these three complementary dimensions, namely inflammation (SII), coagulation (D-dimer), and nutrition (PNI), provides significant incremental predictive value over conventional clinical and radiological tools remains unknown. Furthermore, while machine learning models have been proposed, they often lack bedside interpretability and clinical transparency (17).

To address this gap, we conducted a single-center retrospective cohort study of patients with ruptured intracranial aneurysms undergoing microsurgical clipping or endovascular coiling. We hypothesized that an integrated multidimensional biomarker profile would improve 90-day prognostic accuracy beyond traditional clinical tools. Our objectives were to identify independent predictors of unfavorable 90-day neurological recovery, evaluate the incremental prognostic value of combining SII, D-dimer, and PNI with baseline clinical and radiological factors, and establish a clinically interpretable risk prediction model for early stratification and perioperative care.

## 2 Materials and methods

### 2.1 Patient population and study design

#### 2.1.1 Study design and cohort derivation

This single-center retrospective observational cohort study was conducted at the Department of Neurosurgery, Tianjin Medical University General Hospital. Patients with aSAH consecutively admitted between January 1, 2023, and December 31, 2025, were screened. A total of 267 patients with aSAH were initially identified based on non-contrast cranial computed tomography (CT) demonstrating subarachnoid blood, with the causative intracranial aneurysm verified by CT angiography (CTA), magnetic resonance angiography (MRA), or digital subtraction angiography (DSA).

Consecutive enrollment was adopted to minimize selection bias. No formal sample size calculation was performed *a priori*; however, sample size adequacy was evaluated post -hoc based on events-per-variable (EPV) principles for multivariable prognostic modeling. The follow-up cutoff date was set as March 31, 2026, ensuring that all patients completed the 90-day neurological assessment. The analytic database was locked on April 15, 2026. This study strictly adhered to the Strengthening the Reporting of Observational Studies in Epidemiology (STROBE) and Transparent Reporting of a multivariable prediction model for Individual Prognosis Or Diagnosis (TRIPOD) reporting guidelines.

#### 2.1.2 Ethics and waiver of informed consent

The study was approved by the Ethics Committee of Tianjin Medical University General Hospital (IRB2022-YX-175-01) and was conducted in accordance with the Declaration of Helsinki. As this was a retrospective cohort study utilizing secondary analysis of anonymized routine clinical and laboratory data without modifying patient care or introducing additional interventional risks, the Medical Ethics Committee approved a formal waiver of written informed consent. All personal identifying information was removed prior to analysis.

#### 2.1.3 Multidisciplinary decision-making and standardized perioperative care

Treatment allocation for aneurysm occlusion (microsurgical clipping vs. endovascular coiling) was determined by a multidisciplinary team comprising senior neurosurgeons and neurointerventionalists, considering aneurysm anatomy (location, size, neck width, and arterial branching), hemorrhage extent, clinical grade, patient age, and comorbid conditions.

All patients received standardized neurocritical care according to institutional protocols derived from AHA/ASA and Neurocritical Care Society guidelines:

**Vasospasm and DCI prophylaxis:** oral nimodipine (60 mg every 4 h) was administered routinely upon admission unless severe refractory hypotension intervened.

**Hemodynamic management:** systolic blood pressure (SBP) was strictly maintained below 160 mmHg prior to aneurysm securing; post-securing, normovolemia was maintained, and induced hypertension (SBP target 140–180 mmHg) was initiated if symptomatic vasospasm or DCI developed.

**Cerebrospinal fluid (CSF) management:** external ventricular drainage (EVD) or lumbar drainage was indicated for acute obstructive hydrocephalus (Evans' index >0.3) or severe intraventricular hemorrhage.

**Nutritional and critical care:** enteral nutrition was initiated within 24–48 h of admission for intubated or dysphagic patients; mechanical ventilation and sedation were standardized across ICU stays.

### 2.2 Inclusion and exclusion criteria

#### 2.2.1 Inclusion criteria

- Age ≥18 years;
- Spontaneous aSAH confirmed by non-contrast CT and culprit aneurysm verified by CTA, MRA, or DSA;
- Presentation for the current acute rupture event;
- Successful aneurysm securing via microsurgical clipping or endovascular coiling;
- Complete medical records, admission laboratory panels, and imaging data;
- Completed 90-day neurological follow-up.

#### 2.2.2 Exclusion criteria

- Non-aneurysmal SAH (e.g., traumatic, arteriovenous malformation, cavernous malformation, or dural arteriovenous fistula);
- Absence of aneurysm occlusion therapy;
- Pre-existing severe neurological deficit (premorbid modified Rankin Scale [mRS] \ge 3);
- Terminal illness or active malignant tumor with expected survival < 3 months;
- Key predictor variables missing >20%;
- Lost to 90-day follow-up.

### 2.3 Data collection, variable definitions, and quality control

#### 2.3.1 Data extraction and quality control

Data were extracted independently by two trained researchers using a pre-standardized data collection form from the hospital electronic medical records, PACS, ICU records, and neurosurgical registry. A senior neurosurgeon adjudicated discrepancies. After entry, 20% of cases were randomly re-audited against primary hospital records to ensure data fidelity.

#### 2.3.2 Baseline clinical and imaging parameters

Initial neurological status (GCS, Hunt–Hess grade, WFNS grade) was recorded upon presentation. For patients undergoing emergency intubation or sedation, pre-sedation scores were prioritized. Radiological severity was appraised on initial CT scans by two blinded neurosurgeons, including modified Fisher grade, intraventricular hemorrhage, intracerebral hematoma, and acute hydrocephalus. Acute hydrocephalus was operationally defined as biventricular enlargement (Evans' index >0.3) on initial CT accompanied by clinical signs of elevated intracranial pressure or requirement for CSF diversion.

Aneurysm parameters (maximum diameter, neck width, location, and morphology) were measured using 3D reconstruction workstations. Irregular morphology was defined as lobulated domes, daughter sacs, wall protrusions, or asymmetrical configurations.

#### 2.3.3 Laboratory biomarkers and composite indices

All baseline laboratory parameters were obtained from the first routine venous blood sample collected within 24 h after hospital admission and before initiation of major therapeutic interventions whenever clinically feasible. Potential confounding treatments before blood sampling, including corticosteroid administration, hyperosmolar therapy, and intravenous crystalloid resuscitation, were reviewed from electronic medical records. The median interval between symptom onset and blood sampling was recorded. Laboratory measurements included complete blood count, C-reactive protein (CRP), procalcitonin (PCT), coagulation parameters [prothrombin time (PT), activated partial thromboplastin time (APTT), fibrinogen, and D-dimer], and liver/renal function indicators (serum albumin and total protein).

Composite inflammatory and nutritional indices were calculated as follows:

- **Systemic Immune-Inflammation Index (SII):** platelet count × neutrophil count / lymphocyte count;
- **Systemic Inflammation Response Index (SIRI):** neutrophil count × monocyte count / lymphocyte count;
- **Prognostic Nutritional Index (PNI):** serum albumin (g/L) + 5 × count ( × 10^9^/L);
- **C-Reactive Protein-to-Albumin Ratio (CAR):** CRP (mg/L) / serum albumin (g/L).

#### 2.3.4 Secondary brain injury definitions DCI

Defined according to the 2023 consensus criteria as the occurrence of new focal neurological deficits, a decrease in GCS score ≥2 points, or new cerebral infarction detected by CT/MRI that was not attributable to aneurysm occlusion, surgical injury, hydrocephalus, or metabolic disturbances.

**Symptomatic vasospasm:** Defined as neurological deterioration accompanied by arterial narrowing on CTA/DSA or a mean flow velocity >120 cm/s on transcranial Doppler.

**Rebleeding:** Defined as new intracranial hemorrhage detected on CT with acute clinical deterioration occurring before or after aneurysm occlusion.

### 2.4 Outcome measures

#### 2.4.1 Primary outcome

The primary endpoint was unfavorable neurological recovery at 90 days post-intervention, defined as an mRS score (18) from 3 to 6 (mRS 0–2 denoted favorable recovery). Outcomes were assessed at 90 days via outpatient clinic visits or structured telephone interviews conducted by two outcome assessors who were strictly blinded to admission laboratory markers, imaging details, and perioperative courses.

#### 2.4.2 Secondary outcomes

Secondary endpoints included 90-day all-cause mortality, functional independence (mRS 0–2), complete functional recovery (mRS 0–1), mRS score at hospital discharge, ICU length of stay, and total hospital length of stay.

### 2.5 Statistical analysis

#### 2.5.1 General statistical analysis

Continuous variables were assessed for normality using the Shapiro-Wilk test. Normally distributed variables are presented as mean ± standard deviation (SD) and compared using the independent student's t-test. Non-normally distributed variables are expressed as median (interquartile range, IQR) and analyzed using the Mann–Whitney U- test. Categorical variables are reported as counts (%) and compared using the chi-square test or Fisher's exact test. Standardized mean differences (SMDs) were calculated to assess baseline covariate balance.

#### 2.5.2 Management of missing data

Variables with < 5% missing values were analyzed using complete-case analysis. For variables with 5%−20% missingness, Multiple Imputation by Chained Equations (MICE) was performed to generate 20 imputed datasets. The imputation model included all candidate predictors, baseline auxiliary variables, and the primary outcome (90-day mRS). Estimates from multivariable regression models were pooled using Rubin's rules. Variables with >20% missing data were excluded *a priori*.

#### 2.5.3 Predictor selection, collinearity, and two-step modeling framework

Candidate predictors were screened using univariable logistic regression with a selection threshold of *P* < 0.10 combined with clinical relevance. Treatment modality (microsurgical clipping vs. endovascular coiling) was prespecified as a clinically relevant covariate because treatment selection may be influenced by aneurysm anatomy, morphology, and patient characteristics, and was therefore retained in multivariable models regardless of its crude association with outcome. Multicollinearity was assessed using the variance inflation factor (VIF). No substantial multicollinearity was observed among variables retained in the multivariable models, with all VIF values below 2.5. SIRI was excluded because of strong collinearity with SII (VIF = 6.82). Detailed VIF values are provided in Supplementary Table S1.

To distinguish baseline risk prediction from subsequent in-hospital complications, a two-step modeling framework was applied:

Admission Model (Step 1): Constructed using only variables available within 24 h of admission, including Hunt–Hess grade, modified Fisher grade, acute hydrocephalus, SII, D-dimer, and PNI.

Updated In-Hospital Model (Step 2): Further incorporated the occurrence of in-hospital DCI to evaluate overall prognostic performance.

Multivariable logistic regression models were developed using backward stepwise selection. Effect estimates are presented as odds ratios (ORs) with 95% confidence intervals (CIs).

#### 2.5.4 Nonlinear associations and cutoff determination

Restricted cubic spline (RCS) analyses with 3–4 knots were performed to evaluate potential nonlinear associations between continuous biomarkers (SII, D-dimer, and PNI) and poor 90-day outcomes. Optimal cutoff values for continuous biomarkers were determined using Youden's index (sensitivity + specificity – 1) based on receiver operating characteristic (ROC) curve analysis.

#### 2.5.5 Model performance, internal validation, and overfitting assessment

Discrimination: model discrimination was assessed using the area under the ROC curve (AUC), with differences between models compared using DeLong's- test.

Calibration: model calibration was evaluated using 1,000-resample bootstrap calibration curves, calibration slopes, and the Hosmer–Lemeshow goodness-of-fit test. A bootstrap shrinkage factor was calculated to estimate model optimism.

Clinical Utility: decision curve analysis (DCA) was performed to quantify net clinical benefit across threshold probabilities ranging from 10% to 70%.

#### 2.5.6 Sensitivity and subgroup analyses

Robustness was assessed through sensitivity analyses including complete-case analysis, exclusion of in-hospital mortality cases, alternative definitions of poor functional outcome (mRS ≥2 and mRS ≥4), and penalized logistic regression using least absolute shrinkage and selection operator (LASSO) regression. Interaction analyses were performed to evaluate whether treatment modality (clipping vs. coiling) modified the observed associations. All statistical tests were two-sided, with *P* < 0.05 considered statistically significant. Analyses were conducted using R software (version 4.4.1; R Foundation for Statistical Computing, Vienna, Austria) and IBM SPSS Statistics (version 29.0; IBM Corp., Armonk, NY, USA).

## 3 Results

### 3.1 Study population and missing data profile

A total of 267 patients with confirmed aSAH were screened during the study period. After applying the predefined eligibility criteria, 58 patients were excluded, resulting in a final analytical cohort of 209 patients (Figure 1). During the 90-day follow-up, 143 patients (68.4%) achieved favorable functional outcomes (mRS 0–2), whereas 66 patients (31.6%) experienced unfavorable outcomes (mRS 3–6). 13 patients were excluded because of loss to 90-day follow-up. Baseline characteristics, including age, sex, Hunt–Hess grade, modified Fisher grade, acute hydrocephalus, DCI occurrence, and admission biomarker levels, were comparable between patients included in the final analysis and those lost to follow-up (all *P* > 0.05; Supplementary Table S2), suggesting limited evidence of systematic differences related to follow-up completeness.

**Figure 1:**
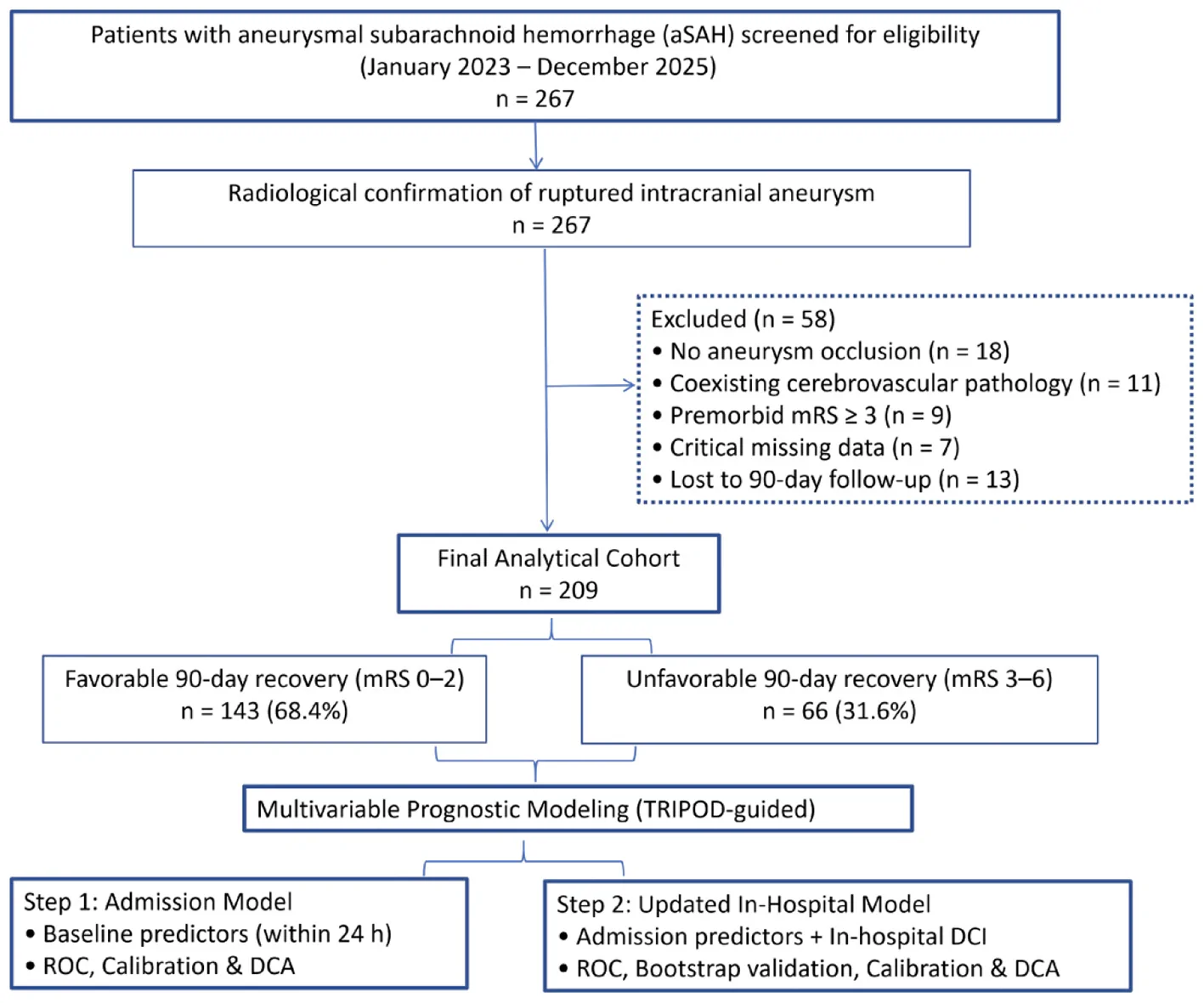
Flow diagram of patient selection and analytical framework. Flowchart depicting the enrollment of patients with aneurysmal subarachnoid hemorrhage (aSAH) according to STROBE guidelines, including exclusion details, cohort derivation (*n* = 209), functional outcome grouping at 90 days (mRS 0–2 vs. mRS 3–6), and the two-step prognostic modeling strategy (Admission Model vs. Updated In-Hospital Model).

Missing data were identified for 12 candidate variables, with proportions ranging from 1.4% (3 / 209 for CRP) to 14.8% (31 / 209 for PCT) (Supplementary Table S3). Variables with < 5% missing values were analyzed using complete-case analysis, while variables with 5%−20% missingness were imputed using Multiple Imputation by Chained Equations (MICE) based on 20 imputed datasets, including the primary outcome. No candidate predictor had >20% missing data.

Baseline demographic and clinical characteristics according to 90-day functional outcomes are presented in Table 1. Compared with patients with favorable outcomes, those with unfavorable outcomes were older (64.5 ± 11.4 vs. 55.8 ± 10.9 years, *P* < 0.001; SMD = 0.78), had higher admission systolic blood pressure (161.8 ± 27.5 vs. 151.6 ± 24.8 mmHg, *P* = 0.008), and more frequently had a history of hypertension (68.2% vs. 48.3%, *P* = 0.008) and previous ischemic stroke (22.7% vs. 8.4%, *P* = 0.003). No significant differences were observed in sex, BMI, smoking status, alcohol consumption, diabetes mellitus, or pre-admission medication use.

**Table 1:** Baseline demographic and clinical characteristics according to 90-day neurological functional outcome.

Variable	Total cohort (*N* = 209)	Good recovery (*n* = 143)	Poor recovery (*n* = 66)	Statistic	*P* value	SMD
Demographics						
Age, years	58.6 ± 11.8	55.8 ± 10.9	64.5± 11.4	*t* = −5.28	< 0.001	0.78
Age ≥65 years, *n* (%)	64 (30.6)	32 (22.4)	32 (48.5)	*χ^2^*= 14.55	< 0.001	0.563
Male sex, *n* (%)	92 (44.0)	60 (42.0)	32 (48.5)	*χ^2^*= 0.77	0.381	0.131
BMI, kg/m^2^	24.5 ± 3.2	24.3 ± 3.1	24.8 ± 3.4	*t* = −1.05	0.294	0.154
BMI ≥28 kg/ m^2^, *n* (%)	28 (13.4)	17 (11.9)	11 (16.7)	*χ^2^*= 0.88	0.348	0.138
Lifestyle factors						
Current smoking, *n* (%)	71 (34.0)	46 (32.2)	25 (37.9)	*χ^2 =^*0.66	0.417	0.120
Alcohol consumption, *n* (%)	60 (28.7)	39 (27.3)	21 (31.8)	*χ^2 =^*0.45	0.501	0.099
Comorbidities						
Hypertension, *n* (%)	114 (54.5)	69 (48.3)	45 (68.2)	*χ^2 =^*7.03	0.008	0.411
Diabetes mellitus, *n* (%)	45 (21.5)	28 (19.6)	17 (25.8)	*χ^2 =^*1.03	0.311	0.148
Coronary artery disease, *n* (%)	27 (12.9)	16 (11.2)	11 (16.7)	*χ^2 =^*1.24	0.266	0.159
Atrial fibrillation, *n* (%)	15 (7.2)	8 (5.6)	7 (10.6)	*χ^2^*= 1.77	0.184	0.185
Previous ischemic stroke, *n* (%)	27 (12.9)	12 (8.4)	15 (22.7)	*χ^2^*= 8.59	0.003	0.402
Previous intracranial hemorrhage, *n* (%)	10 (4.8)	5 (3.5)	5 (7.6)	*χ^2^*= 1.66	0.198	0.181
Chronic kidney disease, *n* (%)	13 (6.2)	7 (4.9)	6 (9.1)	*χ^2^*= 1.35	0.245	0.165
Dyslipidemia, *n* (%)	55 (26.3)	35 (24.5)	20 (30.3)	*χ^2^*= 0.79	0.374	0.131
Preadmission medications						
Antiplatelet therapy, *n* (%)	23 (11.0)	13 (9.1)	10 (15.2)	*χ^2^*= 1.68	0.195	0.189
Anticoagulant therapy, *n* (%)	8 (3.8)	4 (2.8)	4 (6.1)	*χ^2 =^*1.35	0.245	0.16
Statin therapy, *n* (%)	30 (14.4)	18 (12.6)	12 (18.2)	*χ^2 =^*1.16	0.282	0.156
Admission vital signs						
Systolic blood pressure, mmHg	154.8 ± 26.1	151.6 ± 24.8	161.8 ± 27.5	*t*= −2.68	0.008	0.39
Diastolic blood pressure, mmHg	89.1 ± 14.9	87.9 ± 14.3	91.6 ± 15.8	*t* = −1.68	0.095	0.246
Heart rate, beats/min	83.9 ± 14.4	82.7 ± 13.9	86.4 ± 15.1	*t* = −1.75	0.082	0.255
Body temperature, °C	36.8 ± 0.5	36.8 ± 0.5	36.9 ± 0.6	*t* = −1.26	0.209	0.181

Values are expressed as mean ± SD or *n* (%). BMI, body mass index; SD, standard deviation; SMD, standardized mean difference. SMD values >0.10 indicate meaningful baseline imbalance between groups.

### 3.2 Admission neurological severity and radiological characteristics

Initial neurological impairment and radiological bleeding burden were markedly worse in the unfavorable recovery group (Table 2). Patients with poor recovery presented with significantly lower admission GCS scores [median 9 (IQR 6–12) vs. 14 (12–15), *P* < 0.001], and higher proportions of Hunt–Hess grade IV–V (59.1% vs. 18.2%, *P* < 0.001) and WFNS grade IV–V (56.1% vs. 16.8%, *P* < 0.001).

**Table 2:** Initial neurological injury severity and imaging characteristics according to 90-day neurological functional outcome.

Variable	Total cohort (*N* = 209)	Good recovery (*n* = 143)	Poor recovery (n = 66)	Statistic	*P* value	SMD
Clinical severity grades						
Admission GCS score	13 (10–15)	14 (12–15)	9 (6–12)	*Z* = −6.20	< 0.001	1.025
Hunt–Hess grade I–III, *n* (%)	144 (68.9)	117 (81.8)	27 (40.9)	*χ^2^*= 35.27	< 0.001	0.912
Hunt–Hess grade IV–V, *n* (%)	65 (31.1)	26 (18.2)	39 (59.1)	*χ^2^*= 35.27	< 0.001	0.912
WFNS grade I–III, *n* (%)	148 (70.8)	119 (83.2)	29 (43.9)	*χ^2^*= 33.71	< 0.001	0.887
WFNS grade IV–V, *n* (%)	61 (29.2)	24 (16.8)	37 (56.1)	*χ^2^*= 33.71	< 0.001	0.887
Radiological characteristics						
Modified Fisher grade 1–2, *n* (%)	81 (38.8)	70 (49.0)	11 (16.7)	*χ^2^*= 19.83	< 0.001	0.728
Modified Fisher grade 3–4, *n* (%)	128 (61.2)	73 (51.0)	55 (83.3)	*χ^2^*= 19.83	< 0.001	0.728
Intraventricular hemorrhage, *n* (%)	81 (38.8)	40 (28.0)	41 (62.1)	*χ^2^*= 22.19	< 0.001	0.719
Intraparenchymal hematoma, *n* (%)	42 (20.1)	19 (13.3)	23 (34.8)	*χ^2^*= 13.07	< 0.001	0.521
Acute hydrocephalus, *n* (%)	61 (29.2)	27 (18.9)	34 (51.5)	*χ^2 =^*23.27	< 0.001	0.724

Continuous variables are presented as median (interquartile range, IQR); categorical variables are presented as count [percentage, *n* [(%)]. GCS, Glasgow Coma Scale; IQR, interquartile range; SMD, standardized mean difference; WFNS, World Federation of Neurosurgical Societies. SMD values > 0.10 indicate meaningful baseline imbalance between groups.

On admission CT imaging, modified Fisher grade 3–4 was present in 83.3% of unfavorable outcome patients compared to 51.0% in favorable outcome patients (*P* < 0.001). Intraventricular hemorrhage (62.1% vs. 28.0%), intraparenchymal hematoma (34.8% vs. 13.3%), and acute hydrocephalus (51.5% vs. 18.9%) were all significantly more prevalent in the unfavorable group (all *P* < 0.001).

### 3.3 Multidimensional biomarker profiles: inflammation, coagulation, and nutritional reserves

Admission laboratory parameters obtained within 24 h (median interval from ictus to blood sampling: 8.5 h; IQR: 4.2–14.0) demonstrated marked differences in systemic inflammatory status, coagulation activity, and nutritional reserves between patients with favorable and unfavorable outcomes (Figure 2A; detailed values are provided in Supplementary Table S4).

**Figure 2:**
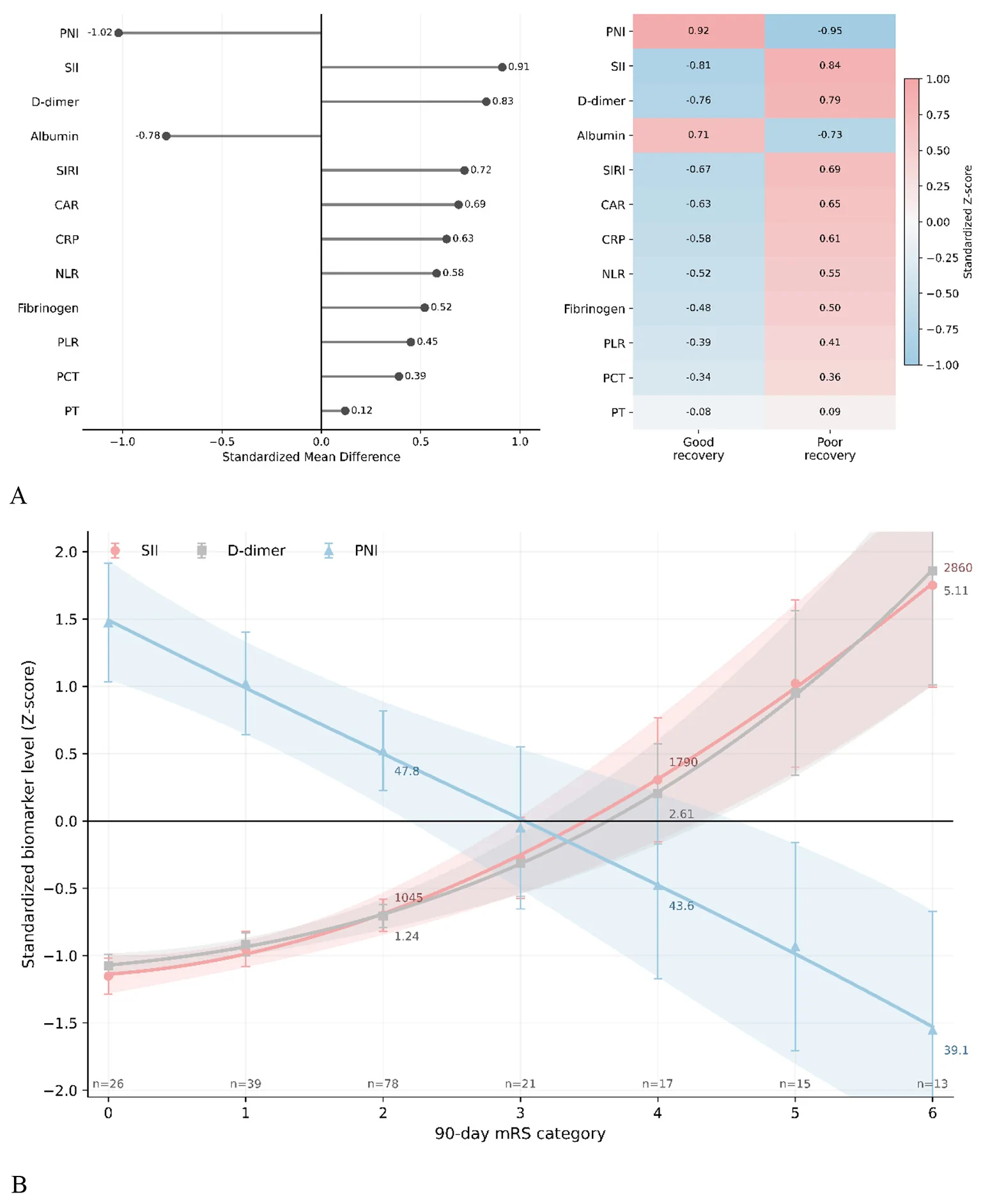
Baseline inflammatory, coagulation, and nutritional status in relation to 90-day neurological recovery**. (A)** Standardized mean differences (SMD, left panel) and *Z*-score normalized heatmaps (right panel) comparing baseline systemic laboratory indicators between favorable (mRS 0–2) and unfavorable (mRS 3–6) recovery groups. Biomarkers are ranked by the absolute magnitude of SMD to highlight indicators with the largest between-group discrimination (complete numerical values provided in Supplementary Table S2). **(B)** Distributional trajectories of standardized *Z*-scores for SII, D-dimer, and PNI across ordinal 90-day mRS categories (grades 0 through 6). Point markers represent group medians with error bars indicating interquartile ranges (IQR); shaded bands represent 95% confidence intervals. Sample size (n) for each mRS category is annotated along the horizontal axis (*P*_*trend*_ < 0.001 across ordinal functional outcome categories for all three biomarkers).

Patients with poor outcomes exhibited significantly higher inflammatory and coagulation-related biomarkers and lower nutritional status. Specifically, SII was significantly elevated in the unfavorable group compared with the favorable group (2,150 × 10^9^/L [IQR: 1,480–2,980] vs. 1,120 × 10^9^/L [IQR: 760–1,540], *P* < 0.001; SMD = 0.91), as was D-dimer (3.82 mg/L [IQR: 2.10–6.45] vs. 1.45 mg/L [IQR: 0.82–2.60], *P* < 0.001; SMD = 0.83). In contrast, PNI was significantly lower among patients with poor outcomes (41.2 ± 4.8 vs. 48.6 ± 4.2, *P* < 0.001; SMD = −1.02).

When analyzed across ordinal 90-day mRS categories (mRS 0–6), SII and D-dimer showed a stepwise increase with worsening functional status, whereas PNI demonstrated a progressive decline (Figure 2B). Furthermore, the proportion of patients with poor 90-day outcomes decreased consistently across PNI quartiles (Q1: < 43.1; Q2: 43.1–46.5; Q3: 46.6–50.2; Q4: >50.2), from 61.5% in Q1 to 9.6% in Q4 (*P*_*trend*_ < 0.001).

### 3.4 Aneurysm features, interventions, and secondary complications

Posterior circulation location (31.8% vs. 14.7%, *P* = 0.004), larger maximum aneurysm diameter 9.7 mm [6.8–13.5] vs.6.8 mm [4.9–9.4], *P* < 0.001), and irregular morphology (56.1% vs. 34.3%, *P* = 0.003) were significantly associated with unfavorable recovery (Table 3). Aneurysm occlusion modality did not significantly differ between clipping (42.4% vs. 46.2%) and coiling (57.6% vs. 53.8%, *P* = 0.609). However, time from onset to treatment was longer in the poor outcome group (28 h [15–46] vs.17 h [9–28], *P* < 0.001).

**Table 3:** Aneurysm characteristics, treatment-related variables, and secondary brain injury events according to 90-day neurological functional outcome.

Variable	Total cohort (*N*=209)	Good recovery (*n*=143)	Poor recovery (*n*=66)	Statistic	P value	SMD
Aneurysm characteristics						
Anterior circulation aneurysm, *n* (%)	167 (79.9)	122 (85.3)	45 (68.2)	*χ^2^*= 8.50	0.004	0.408
Posterior circulation aneurysm, *n* (%)	42 (20.1)	21 (14.7)	21 (31.8)	*χ^2^*= 8.50	0.004	0.408
Maximum aneurysm diameter, mm	7.5 (5.2–10.8)	6.8 (4.9–9.4)	9.7 (6.8–13.5)	Z = −4.18	< 0.001	0.612
Wide-neck aneurysm, *n* (%)	66 (31.6)	41 (28.7)	25 (37.9)	*χ^2^*= 1.79	0.181	0.197
Multiple aneurysms, *n* (%)	49 (23.4)	31 (21.7)	18 (27.3)	*χ^2^*= 0.79	0.374	0.130
Irregular morphology, *n* (%)	86 (41.1)	49 (34.3)	37 (56.1)	*χ^2^*= 8.83	0.003	0.447
Treatment-related variables						
Microsurgical clipping, *n* (%)	94 (45.0)	66 (46.2)	28 (42.4)	*χ^2^*= 0.26	0.609	0.076
Endovascular coiling, *n* (%)	115 (55.0)	77 (53.8)	38 (57.6)	*χ^2^*= 0.26	0.609	0.076
Time from onset to treatment, h	20 (11–32)	17 (9–28)	28 (15–46)	*Z* = −3.61	< 0.001	0.528
External ventricular drainage, *n* (%)	52 (24.9)	24 (16.8)	28 (42.4)	*χ^2^*= 15.78	< 0.001	0.582
Mechanical ventilation, *n* (%)	47 (22.5)	18 (12.6)	29 (43.9)	*χ^2^*= 25.56	< 0.001	0.738
Secondary brain injury and complications						
Delayed cerebral ischemia, *n* (%)	45 (21.5)	18 (12.6)	27 (40.9)	*χ^2^*= 21.55	< 0.001	0.672
Symptomatic vasospasm, *n* (%)	39 (18.7)	17 (11.9)	22 (33.3)	*χ^2^*= 14.24	< 0.001	0.529
New cerebral infarction, *n* (%)	37 (17.7)	14 (9.8)	23 (34.8)	*χ^2^*= 20.03	< 0.001	0.628
Acute hydrocephalus, *n* (%)	48 (23.0)	23 (16.1)	25 (37.9)	*χ^2^*= 12.43	< 0.001	0.502
Seizure, *n* (%)	17 (8.1)	7 (4.9)	10 (15.2)	*χ^2^*= 6.42	0.011	0.347
Rebleeding, *n* (%)	9 (4.3)	4 (2.8)	5 (7.6)	Fisher	0.143	0.218
Pulmonary infection, *n* (%)	53 (25.4)	26 (18.2)	27 (40.9)	*χ^2^*= 12.48	< 0.001	0.508
Hyponatremia, *n* (%)	51 (24.4)	31 (21.7)	20 (30.3)	*χ^2^*= 1.79	0.181	0.198
Recovery quality during hospitalization						
ICU length of stay, d	5 (2–10)	4 (2–7)	11 (6–18)	*Z* = −6.02	< 0.001	0.985
Total hospital length of stay, d	19 (14–27)	17 (13–23)	29 (21–41)	*Z* = −5.68	< 0.001	0.921

Continuous variables are expressed as median (interquartile range, IQR); categorical variables are expressed as count [percentage, *n* (%)]. DCI, delayed cerebral ischemia; ICU, intensive care unit; IQR, interquartile range; SMD, standardized mean difference. SMD values >0.10 indicate meaningful baseline imbalance between groups.

Aneurysm occlusion modality did not significantly differ between clipping and coiling in the overall cohort (45.0% vs. 55.0%, *P* = 0.609). Stratified analyses showed no significant imbalance in treatment modality according to aneurysm location, wide-neck status, irregular morphology, or aneurysm size (Supplementary Table S5).

Secondary brain injury and systemic complications occurred more frequently in the unfavorable group: DCI (40.9% vs. 12.6%, *P* < 0.001), symptomatic vasospasm (33.3% vs. 11.9%, *P* < 0.001), new cerebral infarction (34.8% vs. 9.8%, *P* < 0.001), acute hydrocephalus (37.9% vs. 16.1%, *P* < 0.001), and pulmonary infection (40.9% vs. 18.2%, *P* < 0.001). Consequently, ICU stay (11 d [6–18] vs.4 d [2–7], P < 0.001) and total hospitalization (29 days [21–41] vs. 17 days [13–23], *P* < 0.001) were substantially prolonged.

### 3.5 Predictor selection, collinearity, and dose-response relationships

Univariable logistic regression identified 21 candidate variables associated with poor recovery at *P* < 0.10 (Table 4). Multicollinearity assessment showed that all variables retained in the multivariable models had low collinearity, with VIF values < 2.5 (e.g., Hunt–Hess grade, VIF = 1.62; SII, VIF = 1.84; PNI, VIF = 1.45). SIRI was excluded from subsequent analyses due to substantial collinearity with SII (VIF = 6.82).

**Table 4:** Univariate and multivariable logistic regression analyses for poor neurological recovery at 90 days.

Variable	Univariate OR (95% CI)	*P* value	Admission Model (Step 1)Multivariable OR (95% CI)	*P* value	Updated In-Hospital Model (Step 2)Multivariable OR (95% CI)	*P* value
Demographics & Medical history						
Age (per 10-year increase)	1.38 (1.07–1.79)	0.013	1.22 (0.91–1.65)	0.182	1.19 (0.88–1.62)	0.255
Previous ischemic stroke	2.02 (1.01–4.04)	0.047	1.41 (0.60–3.32)	0.432	1.34 (0.56–3.19)	0.512
Admission neurological severity						
Admission GCS score (per point decrease)	1.20 (1.11–1.32)	< 0.001	1.06 (0.93–1.20)	0.384	1.06 (0.93–1.20)	0.348
Hunt–Hess grade IV–V	6.48 (3.28–12.81)	< 0.001	3.85 (1.84–8.06)	< 0.001	3.12 (1.45–6.71)	0.004
WFNS grade IV–V	6.33 (3.17–12.62)	< 0.001	1.62 (0.70–3.75)	0.261	1.54 (0.65–3.64)	0.327
Radiological characteristics						
Modified Fisher grade 3–4	4.79 (2.18–10.53)	< 0.001	2.76 (1.28–5.95)	0.01	2.41 (1.08–5.39)	0.031
Intraventricular hemorrhage	4.20 (2.18–8.10)	< 0.001	1.45 (0.71–2.98)	0.312	1.37 (0.66–2.84)	0.397
Intraparenchymal hematoma	3.47 (1.63–7.39)	0.001	1.48 (0.63–3.51)	0.37	1.42 (0.59–3.42)	0.435
Acute hydrocephalus	4.56 (2.31–8.99)	< 0.001	2.62 (1.25–5.48)	0.011	2.36 (1.11–5.02)	0.026
Aneurysm & Treatment factors						
Posterior circulation aneurysm	2.71 (1.38–5.33)	0.004	1.81 (0.83–3.92)	0.134	1.72 (0.78–3.80)	0.179
Irregular morphology	2.45 (1.34–4.48)	0.004	1.55 (0.80–3.01)	0.192	1.49 (0.76–2.91)	0.246
Time from onset to treatment (per 12 h)	1.29 (1.10–1.53)	0.003	1.16 (0.98–1.38)	0.082	1.18 (0.99–1.41)	0.064
Mechanical ventilation	5.43 (2.76–10.67)	< 0.001	1.78 (0.80–3.95)	0.158	1.69 (0.75–3.80)	0.206
In-hospital secondary brain injury						
Delayed cerebral ischemia (DCI)	4.81 (2.45–9.43)	< 0.001	—^*^	—^*^	3.58 (1.62–7.91)	0.002
Symptomatic vasospasm	3.70 (1.82–7.54)	< 0.001	—^*^	—^*^	1.51 (0.62–3.65)	0.361
New cerebral infarction	4.93 (2.33–10.41)	< 0.001	—^*^	—^*^	1.83 (0.78–4.28)	0.164
Laboratory biomarkers						
SII (per 500-unit increase)	1.42 (1.25–1.62)	< 0.001	1.32 (1.14–1.53)	< 0.001	1.28 (1.10–1.49)	0.001
SIRI (per 1-unit increase)	1.23 (1.08–1.40)	0.002	—^**^	—^**^	—^**^	—^**^
CAR (per 1-unit increase)	1.17 (1.05–1.31)	0.006	1.14 (0.97–1.33)	0.118	1.13 (0.96–1.32)	0.139
D-dimer (per 1 mg/L increase)	1.34 (1.19–1.51)	< 0.001	1.22 (1.07–1.39)	0.003	1.19 (1.05–1.36)	0.011
PNI (per 5-point increase)	0.62 (0.52–0.74)	< 0.001	0.68 (0.55–0.84)	< 0.001	0.71 (0.57–0.88)	0.003
Treatment modality (coiling vs clipping)	1.16 (0.68–1.98)	0.609	1.12 (0.61–2.06)	0.72	1.10 (0.59–2.05)	0.76

Bold values indicate statistically significant independent predictors (*P* < 0.05).

CAR, C-reactive protein-to-albumin ratio; CI, confidence interval; DCI, delayed cerebral ischemia; GCS, Glasgow Coma Scale; OR, odds ratio; PNI, prognostic nutritional index; SII, systemic immune-inflammation index; SIRI, systemic inflammation response index; WFNS, World Federation of Neurosurgical Societies.

^*^DCI and in-hospital secondary brain injury events were intentionally excluded from the Admission Model (Step 1) to construct a strict early prognostic tool available within 24 h of admission.

^**^ SIRI was excluded from the multivariable models because of substantial collinearity with SII (VIF = 6.82).

SIRI was excluded from multivariable models due to high collinearity with SII [variance inflation factor (VIF) = 6.82]. All retained predictors exhibited excellent collinearity profiles with VIF values < 2.5 (e.g., Hunt–Hess VIF = 1.62, modified Fisher VIF = 1.48, acute hydrocephalus VIF = 1.35, DCI VIF = 1.52, SII VIF = 1.84, D-dimer VIF = 1.61, PNI VIF = 1.45).

Restricted cubic spline analysis demonstrated a significant nonlinear association between PNI and unfavorable neurological recovery (Poverall < 0.001; Pnon-linear = 0.028). The risk of poor recovery increased substantially when PNI decreased below approximately 45 points. ROC analysis identified an optimal PNI cutoff of 45.2, yielding a sensitivity of 80.3% and specificity of 76.2% (Supplementary Figure S1).

#### 3.5.1 Association between admission PNI and in-hospital nutritional trajectory

Because nutritional reserve may influence recovery after acute neurological injury, we further explored the association between admission PNI and subsequent nutritional support intensity as well as changes in serum albumin levels during hospitalization.

Patients were stratified according to the previously identified optimal PNI cutoff of 45.2. Compared with patients with PNI ≥45.2, those with lower admission PNI (< 45.2) demonstrated greater nutritional vulnerability during hospitalization. Patients with lower PNI required longer enteral nutritional support and exhibited greater cumulative nutritional intervention requirements. Specifically, the duration of enteral feeding was significantly longer in patients with PNI < 45.2 than in those with PNI ≥45.2 [median 12 (IQR: 8–18) days vs. 8 (IQR: 5–12) days, P < 0.001]. Furthermore, patients with lower admission PNI experienced a greater decline in serum albumin concentration from admission to discharge (−6.8 ± 3.1 g/L vs. −3.2 ± 2.5 g/L, *P* < 0.001), suggesting accelerated nutritional depletion during the acute recovery phase.

In multivariable linear regression analysis adjusted for age, Hunt–Hess grade IV–V, modified Fisher grade 3–4, DCI occurrence, and ICU length of stay, lower admission PNI remained independently associated with greater in-hospital albumin decline (β = −0.42, 95% CI: −0.61 to −0.23, *P* < 0.001).

Furthermore, logistic regression analysis demonstrated that lower admission PNI was independently associated with prolonged enteral nutrition requirement (>14 days) after adjustment for potential clinical confounders (adjusted OR=2.31, 95% CI: 1.34–3.98, *P* = 0.003) (Supplementary Figure S2).

The association between longer onset-to-treatment time and poor recovery was attenuated after adjustment for baseline neurological severity, suggesting that treatment delay may partly reflect initial disease severity rather than an independent causal pathway. A supplementary model retaining onset-to-treatment time demonstrated consistent effect estimates (Supplementary Table S6).

### 3.6 Two-step multivariable prediction models

To avoid temporal overlap between baseline risk factors and post-admission complications, multivariable logistic regression was performed using a two-step modeling framework (Figure 3).

**Figure 3:**
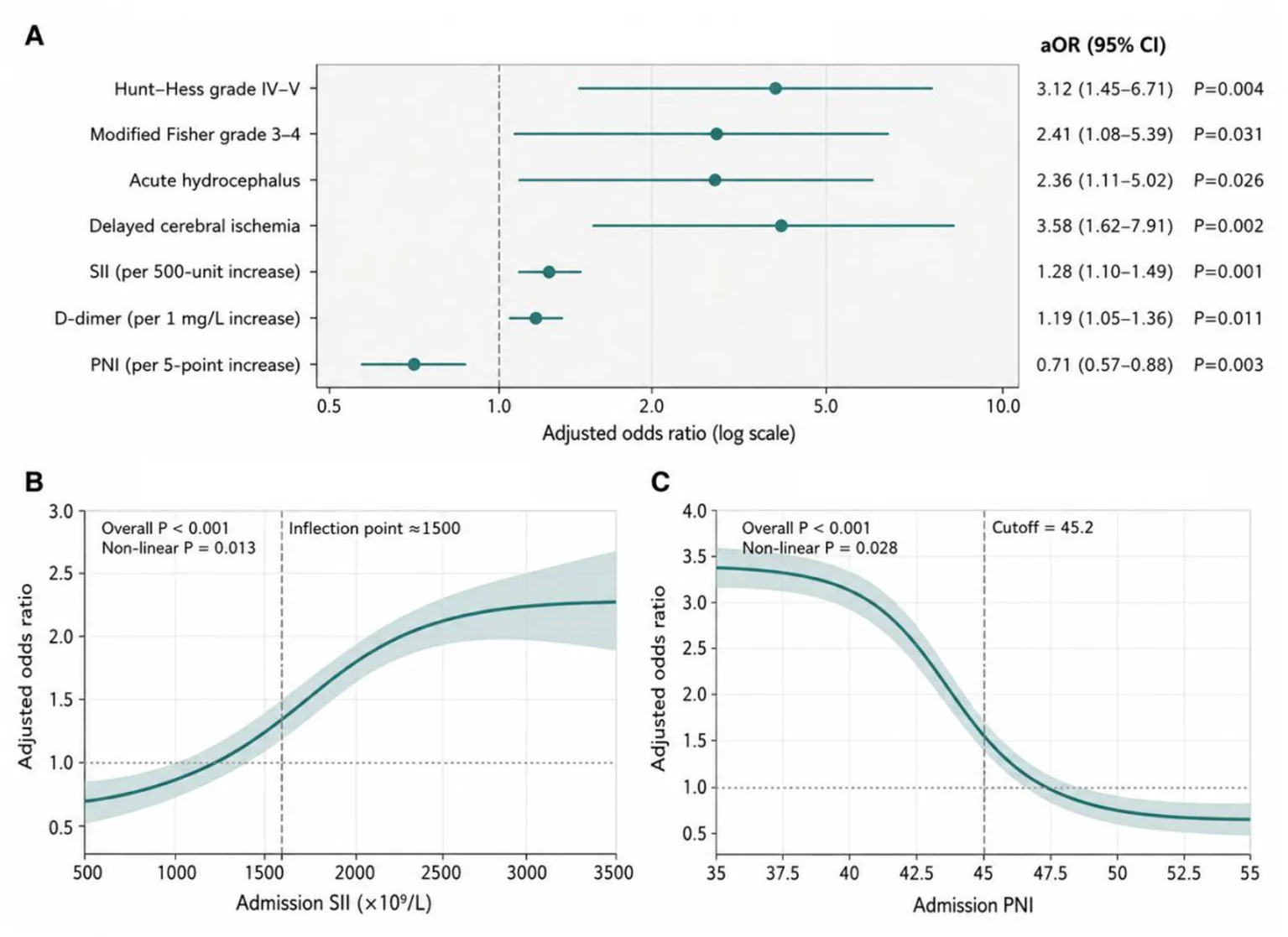
Independent prognostic factors and non-linear dose-response relationship analysis**. (A)** Forest plot displaying adjusted odds ratios (aOR) and 95% confidence intervals (CI) for independent predictors derived from the multivariable logistic regression framework (Step 2: Updated In-Hospital Model). **(B)** Restricted cubic spline (RCS) analysis showing the non-linear dose-response relationship between admission SII and unfavorable 90-day recovery (*P*_*overall*_ < 0.001, *P*_*non*−*linear*_ = 0.013), with an inflection point identified at ≈1500 × 10^9^L. **(C)** RCS analysis evaluating the inverse non-linear relationship between admission PNI and poor functional recovery (*P*_*overall*_ < 0.001, *P*_*non*−*linear*_= 0.028), demonstrating an accelerated risk of poor outcome when PNI falls below the optimal clinical threshold of 45.2. Solid dark lines represent adjusted ORs, shaded bands indicate 95% CIs, and vertical dashed lines mark key clinical cutoff points.

**Step 1: Admission model (Variables available within 24 h)** Among 66 outcome events and six candidate predictors (EPV = 11.0), independent admission predictors were identified as follows:

Hunt–Hess grade IV–V: adjusted OR (aOR) 3.85 (95% CI: 1.84–8.06, *P* < 0.001)

Modified Fisher grade 3–4: aOR 2.76 (95% CI: 1.28–5.95, P = 0.010)

Acute hydrocephalus: aOR 2.62 (95% CI: 1.25–5.48, *P* = 0.011)

SII (per 500-unit increase): aOR 1.32 (95% CI: 1.14–1.53, *P* < 0.001)

D-dimer (per 1 mg/L increase): aOR 1.22 (95% CI: 1.07–1.39, *P* = 0.003)

PNI (per 5-point increase): aOR 0.68 (95% CI: 0.55–0.84, *P* < 0.001)

**Step 2: Updated in-hospital model (Incorporating DCI)**

The updated in-hospital model incorporated DCI, an in-hospital event occurring after admission, to evaluate whether secondary complications provided additional prognostic information beyond baseline predictors. After adding DCI to the model (EPV = 9.4), DCI emerged as an independent predictor of unfavorable neurological recovery (aOR 3.58, 95% CI: 1.62–7.91, *P* = 0.002). Other independent predictors remained significant, including Hunt–Hess grade IV–V (aOR 3.12), modified Fisher grade 3–4 (aOR 2.41), acute hydrocephalus (aOR 2.36), SII (aOR 1.28 per 500-unit increase), D-dimer (aOR 1.19 per 1 mg/L increase), and PNI (aOR 0.71 per 5-point increase) (Table 4).

Treatment modality was retained as an adjustment covariate because of potential confounding by indication but was not independently associated with unfavorable recovery (coiling vs. clipping: aOR=1.10, 95% CI: 0.59–2.05, *P* = 0.760).

The updated in-hospital model demonstrated an apparent AUC of 0.86 (95% CI: 0.80–0.91), with a sensitivity of 81.8% and specificity of 78.3% (Figure 4A). Internal validation using 1,000 bootstrap resamples yielded an optimism-corrected AUC of 0.83 and a shrinkage factor of 0.91, indicating limited optimism. Calibration analysis demonstrated good agreement between predicted and observed risks (calibration slope = 0.94; Hosmer–Lemeshow χ^2^ = 6.18, *P* = 0.62; Figure 4B).

**Figure 4:**
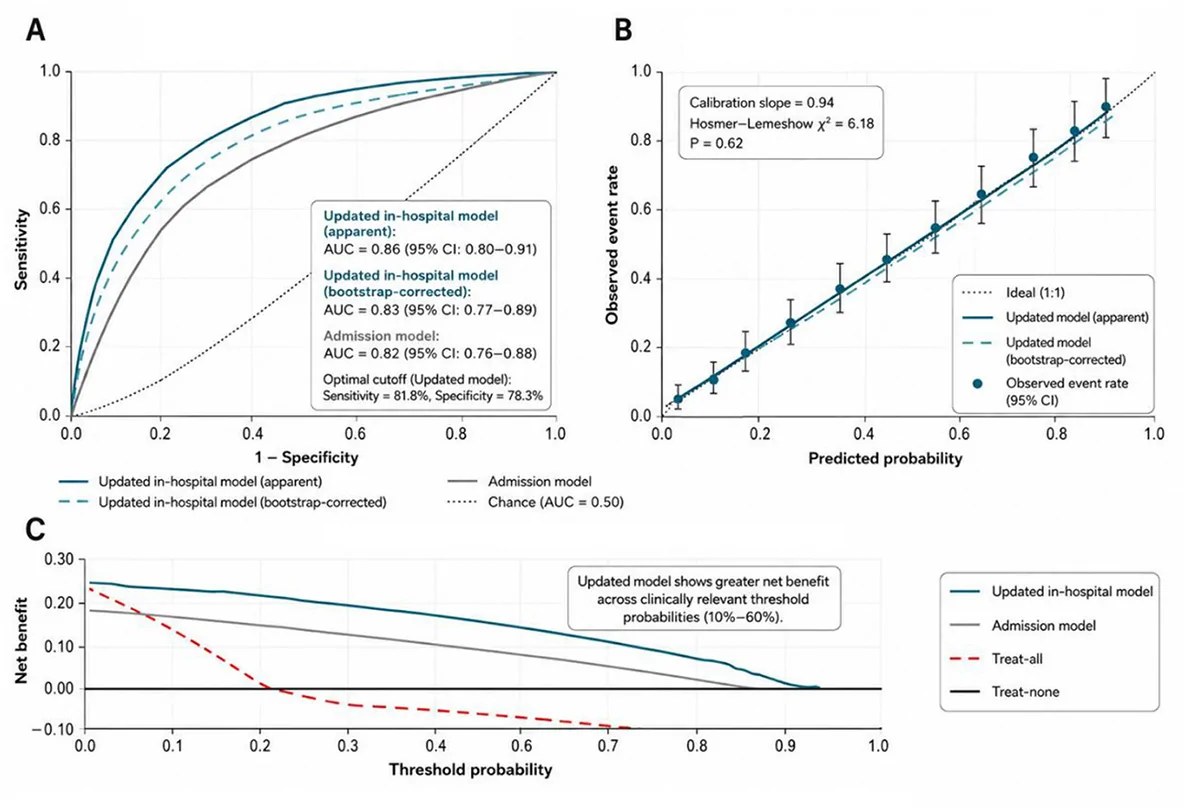
Discrimination, internal validation, calibration, and clinical utility of the two-step prognostic models. **(A)** Receiver operating characteristic (ROC) curves comparing the discrimination performance of the admission model and updated in-hospital model for predicting unfavorable 90-day functional outcomes. The updated in-hospital model demonstrated improved discrimination compared with the admission model, with an apparent area under the curve (AUC) of 0.86 (95% CI: 0.80–0.91) and an optimism-corrected AUC of 0.83 after 1,000-bootstrap internal validation. The admission model showed an AUC of 0.82 (95% CI: 0.76–0.88). At the optimal cutoff, the updated model achieved a sensitivity of 81.8% and specificity of 78.3%. **(B)** Calibration plot of the updated in-hospital model for predicting unfavorable 90-day functional outcomes. The dotted line represents ideal calibration, whereas the solid and dashed curves indicate apparent and bootstrap-corrected calibration, respectively. The model showed good calibration performance, with a calibration slope of 0.94 and a Hosmer–Lemeshow goodness-of-fit test result of χ^2^ = 6.18 (*P* = 0.62). Points represent observed event rates with 95% confidence intervals across risk-stratified deciles. **(C)** Decision curve analysis demonstrating the clinical utility of the admission and updated in-hospital models. The updated in-hospital model provided greater net benefit across clinically relevant threshold probabilities (10%−60%) compared with the admission model, treat-all strategy, and treat-none strategy. ROC, receiver operating characteristic; AUC, area under the curve; CI, confidence interval; DCA, decision curve analysis.

For comparison, the Admission Model demonstrated good discrimination, with an AUC of 0.82 (95% CI: 0.76–0.88). Bootstrap internal validation yielded an optimism-corrected AUC of 0.80. Calibration analysis showed good agreement between predicted and observed probabilities (calibration slope = 0.91; Hosmer–Lemeshow χ^2^ = 7.12, *P* = 0.52). Decision curve analysis indicated a positive net clinical benefit across threshold probabilities of 10%−60% compared with treat-all and treat-none strategies (Figure 4C).

### 3.7 Incremental value of biomarkers and clinical decision analysis

Adding the triad of biological markers (SII, D-dimer, PNI) to a baseline clinical-radiological model (Hunt–Hess, modified Fisher, acute hydrocephalus, DCI) significantly improved overall discrimination, increasing the AUC from 0.78 (95% CI: 0.72–0.84) to 0.86 (95% CI: 0.80–0.91) (DeLong- test *P* = 0.011; Figure 5A).

**Figure 5:**
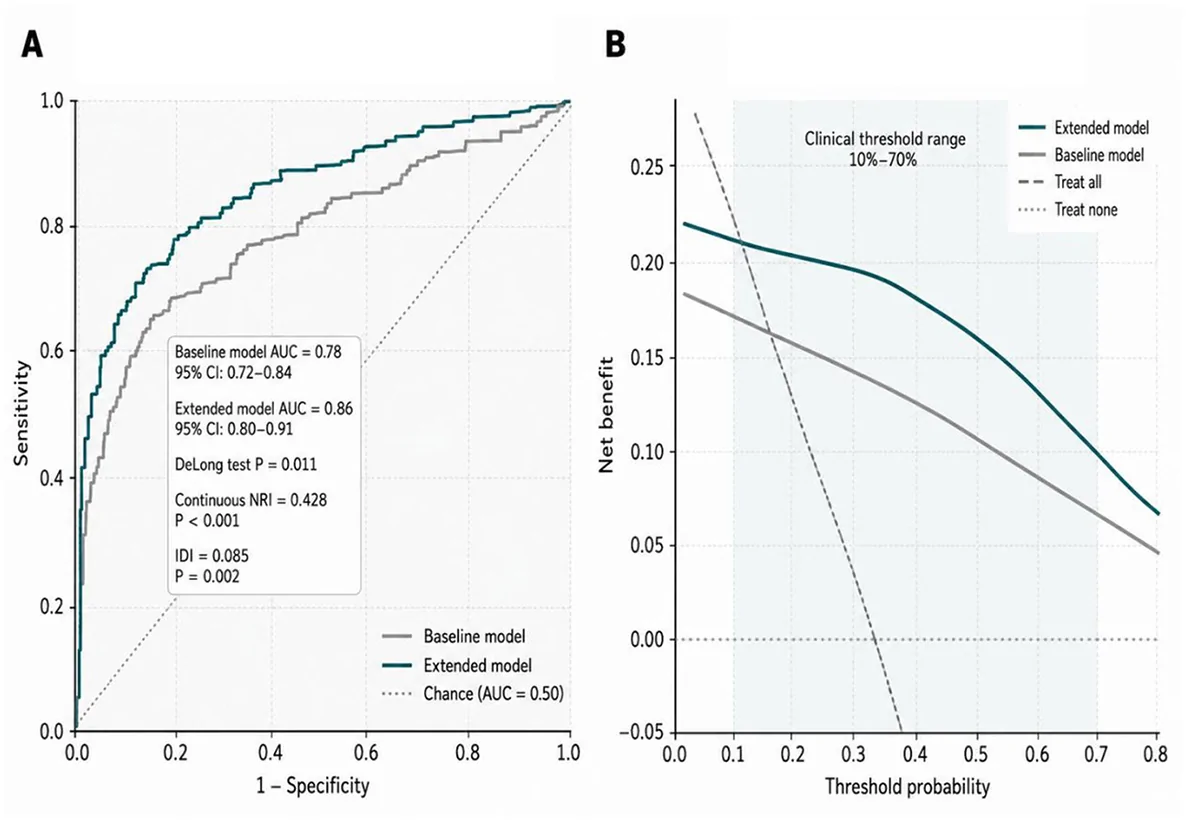
Incremental predictive performance and clinical utility of the inflammatory, coagulation, and nutritional biomarker triad. **(A)** Receiver operating characteristic (ROC) curves comparing the baseline model (Hunt–Hess grade, modified Fisher grade, acute hydrocephalus, and DCI; AUC = 0.78, 95% CI: 0.72–0.84) with the extended model incorporating SII, D-dimer, and PNI (AUC = 0.86, 95% CI: 0.80–0.91). Addition of the biomarker triad significantly improved model discrimination (DeLong -test, P = 0.011), with further improvement supported by continuous net reclassification improvement (NRI = 0.428, *P* < 0.001) and integrated discrimination improvement (IDI = 0.085, *P* = 0.002). **(B)** Decision curve analysis evaluating the net clinical benefit of the extended model compared with the baseline model across threshold probabilities of 0%−80%. The extended model demonstrated consistently greater clinical utility than the baseline model and the conventional “treat-all” and “treat-none” strategies within the clinically relevant threshold range of 10%−70%.

DCA showed that the extended model provided higher net clinical benefit than both the baseline model and “treat-all/treat-none” strategies across decision threshold probabilities of 10% to 70% (Figure 5B).

### 3.8 Robustness, subgroup, and secondary outcome analyses

Sensitivity analyses involving complete cases (*n* = 188), exclusion of in-hospital deaths (*n* = 183), and alternative mRS thresholds (≥2 or ≥4) consistently demonstrated the robustness of the associations between SII, D-dimer, PNI, and clinical outcomes (Table 5). Subgroup analyses stratified by treatment modality (clipping, *n* = 94; coiling, *n* = 115) revealed no significant interactions for any predictor (all *P*_*interaction*_ >0.60).

**Table 5:** Sensitivity and subgroup analyses for independent predictors of unfavorable 90-day neurological recovery.

Variable	Primary Analysis(*N* = 209)	Complete-Case Analysis(*n* = 188)	Excluding In-Hospital Deaths(*n* = 183)	Outcome Redefined as mRS ≥2(*N* = 209)	Outcome Redefined as mRS ≥4(*N* = 209)	Clipping Subgroup(*n* = 94)	Endovascular Subgroup(*n* = 115)	*P* for Interaction^*^
Clinical & Radiological Predictors								
Hunt–Hess grade IV–V	3.12 (1.45–6.71)	3.05 (1.39–6.68)	2.94 (1.32–6.52)	2.46 (1.21–5.01)	3.88 (1.61–9.36)	3.28 (1.21–8.90)	2.91 (1.08–7.86)	0.74
Modified Fisher grade 3–4	2.41 (1.08–5.39)	2.35 (1.03–5.34)	2.26 (0.98–5.21)	1.89 (0.94–3.80)	3.16 (1.28–7.82)	2.52 (0.94–6.77)	2.28 (0.86–6.02)	0.83
Acute hydrocephalus	2.36 (1.11–5.02)	2.27 (1.05–4.92)	2.19 (1.01–4.76)	1.94 (0.98–3.85)	3.21 (1.36–7.59)	2.42 (0.98–5.96)	2.31 (0.95–5.61)	0.88
Delayed cerebral ischemia (DCI)	3.58 (1.62–7.91)	3.41 (1.53–7.63)	3.22 (1.41–7.33)	2.76 (1.31–5.81)	4.42 (1.78–10.96)	3.67 (1.29–10.47)	3.39 (1.18–9.72)	0.93
Biological Biomarkers								
SII (per 500-unit increase)	1.28 (1.10–1.49)	1.26 (1.08–1.48)	1.24 (1.05–1.47)	1.21 (1.05–1.39)	1.36 (1.13–1.64)	1.31 (1.05–1.64)	1.27 (1.03–1.58)	0.81
D-dimer (per 1 mg/L increase)	1.19 (1.05–1.36)	1.17 (1.03–1.34)	1.18 (1.03–1.35)	1.12 (0.99–1.27)	1.31 (1.10–1.56)	1.22 (1.01–1.47)	1.16 (0.99–1.37)	0.67
PNI (per 5-point increase)	0.71 (0.57–0.88)	0.73 (0.58–0.91)	0.74 (0.59–0.93)	0.78 (0.64–0.95)	0.63 (0.48–0.83)	0.69 (0.49–0.96)	0.72 (0.54–0.95)	0.89

Data are presented as adjusted Odds Ratios (OR) with 95% Confidence Intervals (CI) derived from multivariable logistic regression. Bold values indicate statistical significance (*P* < 0.05). CI, confidence interval; mRS, modified Rankin Scale; OR, odds ratio; PNI, prognostic nutritional index; SII, systemic immune-inflammation index. All models were adjusted for baseline demographics (age, previous stroke) and radiological/clinical severity parameters.

For secondary outcomes (Figure 6A), elevated admission SII and D-dimer levels were independently associated with increased 90-day mortality and reduced likelihood of functional independence (mRS 0–2), with adjusted ORs of 1.42 and 1.31, respectively. In contrast, higher PNI was independently associated with lower mortality risk (aOR = 0.66, 95% CI: 0.51–0.86) and greater likelihood of complete recovery (mRS 0–1; aOR = 1.41, 95% CI: 1.15–1.73). Secondary brain injury events, including DCI and new cerebral infarction, were significantly associated with prolonged ICU and hospital stays (*P* < 0.001; Figure 6B).

**Figure 6:**
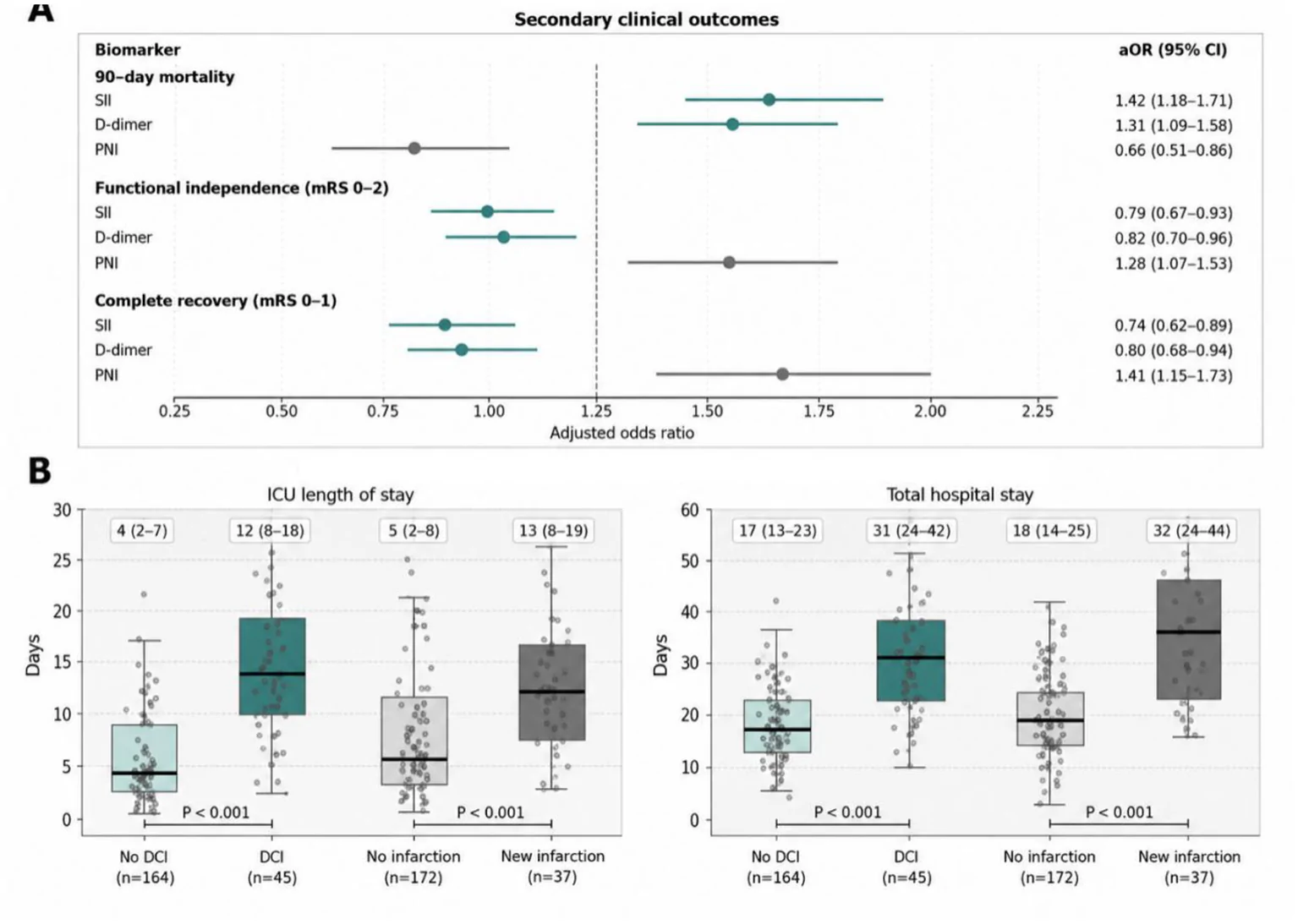
Secondary clinical outcomes and healthcare resource utilization. **(A)** Forest plot showing multivariable-adjusted odds ratios (aORs) with 95% confidence intervals (CIs) for admission SII, D-dimer, and PNI in relation to secondary clinical outcomes, including 90-day all-cause mortality, functional independence (mRS 0–2), and complete recovery (mRS 0–1). All models were adjusted for age, previous stroke history, Hunt–Hess grade, and modified Fisher grade. **(B)** Box plots illustrating intensive care unit (ICU) and total hospital length of stay according to secondary brain injury events, including delayed cerebral ischemia (DCI; no DCI, *n* = 164; DCI*, n* = 45) and new cerebral infarction (no infarction, *n* = 172; new infarction, *n* = 37). Horizontal lines within boxes indicate median values, with annotations showing median (interquartile range, IQR) lengths of stay in days. Individual patient values are displayed as jittered points. All comparisons were performed using the Mann–Whitney U- test (*P* < 0.001).

Penalized logistic regression using LASSO regression demonstrated consistent predictor selection with the primary multivariable model. The retained predictors included Hunt–Hess grade IV–V, modified Fisher grade 3–4, acute hydrocephalus, DCI, SII, D-dimer, and PNI, supporting the stability of the final model despite the moderate events-per-variable ratio. Ten-fold cross-validation identified an optimal lambda value of 0.032, and no additional predictors beyond those included in the primary model were selected.

#### 3.8.1 Sensitivity analysis according to timing of biomarker measurement

To evaluate whether the timing of biomarker measurement influenced the observed associations, a sensitivity analysis was performed by stratifying patients according to the interval between symptom onset and the first blood sampling ( ≤ 6 h vs. 6–24 h). The associations of SII, D-dimer, and PNI with unfavorable neurological recovery remained directionally consistent across both subgroups, with no significant interaction effects observed (all P for interaction >0.10), suggesting that the prognostic associations were not substantially modified by the timing of baseline biomarker assessment.

Among patients with blood samples obtained within 6 h after symptom onset, elevated SII remained independently associated with poor recovery [adjusted OR (aOR) = 1.31, 95% CI: 1.10–1.56, *P* = 0.003], as did higher D-dimer levels (aOR=1.20, 95% CI: 1.02–1.42, *P* = 0.028). Higher PNI remained protective against unfavorable recovery (aOR = 0.69, 95% CI: 0.53–0.89, *P* = 0.006).

In patients whose blood samples were obtained between 6 and 24 h after symptom onset, the associations remained stable, with SII (aOR = 1.27, 95% CI: 1.09–1.48, *P* = 0.002), D-dimer (aOR = 1.18, 95% CI: 1.03–1.35, *P* = 0.016), and PNI (aOR = 0.72, 95% CI: 0.58–0.90, *P* = 0.004) showing similar associations with unfavorable neurological recovery (Supplementary Table S7).

A worst-case sensitivity analysis was performed by assigning all patients lost to follow-up as having unfavorable neurological outcomes (mRS 3–6). Under this conservative assumption, the associations between admission biomarkers and poor neurological recovery remained consistent with the primary analysis. The adjusted effect estimates were minimally changed for SII (aOR = 1.29, 95% CI: 1.12–1.49, *P* < 0.001), D-dimer (aOR=1.20, 95% CI: 1.06–1.37, P=0.004), and PNI (aOR=0.70, 95% CI: 0.57–0.86, *P* < 0.001), supporting the robustness of the findings against potential outcome misclassification caused by missing follow-up (Supplementary Table S8).

## 4 Discussion

In this retrospective cohort study of patients with ruptured intracranial aneurysms, we found that high Hunt–Hess grade (IV–V), high modified Fisher grade (3–4), acute hydrocephalus, in-hospital DCI, elevated SII, elevated D-dimer, and low PNI were independently associated with unfavorable 90-day neurological recovery. The addition of these three biological markers to conventional clinical and radiological scores significantly improved model discrimination, calibration, and net clinical benefit. These findings suggest that a multidimensional prognostic framework integrating inflammatory, coagulation, and nutritional parameters can refine risk stratification after aneurysm repair.

The prognostic importance of initial hemorrhage severity and neurological impairment was reaffirmed in our cohort. Hunt–Hess grade IV–V and modified Fisher grade 3–4 remained robust independent predictors, consistent with extensive evidence linking severe early brain injury and extensive subarachnoid blood to microcirculatory failure, cerebral edema, and subsequent DCI (19, 20). Acute hydrocephalus independently increased the odds of poor recovery, likely through impaired cerebrospinal fluid circulation and elevated intracranial pressure. Rather than acting as isolated variables, these clinical and radiological scores reflect both acute metabolic disruption of cerebral tissue and susceptibility to secondary complications.

To address the conceptual challenge of using in-hospital events for early prognostication, we adopted a two-step modeling framework. The admission model, which incorporated Hunt–Hess grade, modified Fisher grade, acute hydrocephalus, SII, D-dimer, and PNI, achieved good discrimination (AUC 0.82) within 24 h of arrival, providing clinicians with an immediate tool for early risk triage. When DCI occurred as a secondary event, the updated in-hospital model further improved performance (AUC 0.86), indicating that dynamic monitoring of delayed complications adds prognostic value. This staged approach resolves the temporal ambiguity between baseline variables and post-admission events and supports a dynamic risk monitoring strategy throughout hospitalization.

Systemic inflammation and coagulation activation were strongly correlated with functional outcomes. SII integrates neutrophil-driven inflammatory cascades, platelet-mediated microthrombosis, and lymphocyte-related immune exhaustion (21, 22). The restricted cubic spline analysis revealed a marked non-linear dose-response pattern, with the risk of poor recovery escalating rapidly when SII exceeded approximately 1500 × 10^9^/L. Elevated admission D-dimer was independently associated with poor functional status and 90-day mortality, corroborating prior evidence that early fibrinolysis and intravascular microthrombosis contribute to secondary microcirculatory failure (23). Although admission measurements provide a useful baseline snapshot, they represent a single time point in a rapidly evolving process. Dynamic changes in these markers during hospitalization may better capture persistent inflammatory activation or resolution, and future studies incorporating serial measurements are warranted to determine whether longitudinal trajectories outperform single assessments.

The nutritional dimension, as reflected by PNI, deserves particular attention. PNI combines serum albumin and lymphocyte count, serving as a proxy for both metabolic reserve and immunocompetence. We observed an inverse association in which each 5-point increase in PNI conferred a 29% reduction in the risk of unfavorable outcome, with a non-linear relationship identifying a clinical threshold around 45.2. Patients with admission PNI below this cutoff experienced greater albumin decline and required longer enteral nutritional support during hospitalization, suggesting that baseline nutritional vulnerability is associated with accelerated metabolic stress after aneurysm rupture. Acute brain injury frequently induces hypercatabolism, hypoalbuminemia, and immunoparalysis, which collectively impair neuronal repair and increase susceptibility to infections (24, 25). From a translational perspective, early identification of patients with low PNI on admission may facilitate targeted nutritional screening, structured early enteral nutrition protocols, and close monitoring of albumin trajectories during intensive care. Whether such nutritional interventions can directly modify neurological outcomes requires prospective investigation, but our findings support PNI as a potentially useful marker for identifying patients at higher metabolic risk.

Our extended prediction model demonstrated strong discrimination, bootstrap-corrected calibration (calibration slope 0.94), and consistent net benefit across clinically relevant threshold probabilities (10% to 70%). Unlike opaque machine learning algorithms, the logistic regression structure maintains full interpretability, allowing clinicians to weigh individual risk factors transparently at the bedside (26, 27). Sensitivity analyses using complete-case analysis, exclusion of in-hospital deaths, alternative mRS cutoffs, and LASSO penalized regression all confirmed the robustness of the observed associations. Subgroup analyses stratified by treatment modality showed no significant interaction, further supporting the stability of these systemic biomarkers across clipping and coiling populations.

Several limitations warrant consideration. First, the retrospective single-center design may introduce selection bias and residual confounding from unmeasured factors such as fluid balance, sedation protocols, and intraoperative hemodynamics. The observed associations should therefore be interpreted as correlational rather than causal. Second, although only 13 patients (4.9%) were lost to 90-day follow-up and baseline characteristics were comparable between included and excluded patients, we cannot completely exclude the possibility of survivorship bias. Third, biomarkers were measured only at admission; repeated measurements might provide additional prognostic insight by capturing dynamic changes in inflammation, coagulation, and nutrition. Fourth, although we performed rigorous internal validation using 1,000 bootstrap resamples, external validation in independent cohorts is essential before clinical implementation. We note that a fully comparable external cohort from our collaborating institution was not available for the current analysis because of differences in data availability and case selection during the study period. Therefore, our model should currently be regarded as exploratory and hypothesis-generating rather than a definitive clinical decision tool. Finally, the relatively modest number of outcome events (66 events) yielded an events-per-variable ratio of approximately 9.4 for the updated in-hospital model, and some optimism or overfitting cannot be entirely excluded. Future multicenter studies with larger sample sizes and independent validation cohorts are needed to confirm generalizability and clinical utility.

In conclusion, unfavorable 90-day functional recovery after ruptured intracranial aneurysm repair is associated with initial hemorrhage severity, acute hydrocephalus, in-hospital secondary brain injury, and systemic biological imbalances. Beyond conventional clinical and radiological grading, admission SII, D-dimer, and PNI reflect complementary pathophysiological dimensions encompassing inflammatory-immune activation, prothrombotic tendency, and metabolic reserve. Integrating these routinely available, low-cost biomarkers into a transparent prediction model significantly improves prognostic discrimination and net clinical benefit over traditional tools alone. Future prospective, multicenter studies adhering to TRIPOD guidelines are warranted to externally validate this framework and to evaluate whether targeted perioperative interventions, including early nutritional optimization and anti-inflammatory strategies, can improve long-term neurological recovery for aSAH patients.

## Data Availability

The raw data supporting the conclusions of this article will be made available by the authors, without undue reservation.

## References

[1] Lv B Lan JX Si YF Ren YF Li MY Guo FF . Epidemiological trends of subarachnoid hemorrhage at global, regional, and national level: a trend analysis study from 1990 to 2021. Mil Med Res. (2024) 11:46. doi: 10.1186/s40779-024-00551-6 38992778 PMC11241879

[2] Hoh BL Ko NU Amin-Hanjani S Chou SH-Y Cruz-Flores S Dangayach NS . 2023 guideline for the Management of patients with aneurysmal subarachnoid hemorrhage: a guideline from the American heart association/American stroke association. Stroke. (2023) 54:e314–70. doi: 10.1161/STR.0000000000000436 37212182

[3] Treggiari MM Rabinstein AA Busl KM Caylor MM Citerio G Deem S . Guidelines for the neurocritical care management of aneurysmal subarachnoid hemorrhage. Neurocrit Care. (2023) 39:1–28. doi: 10.1007/s12028-023-01713-5 37202712

[4] Thilak S Brown P Whitehouse T Gautam N Lawrence E Ahmed Z . Diagnosis and management of subarachnoid hemorrhage. Nat Commun. (2024) 15:1850. doi: 10.1038/s41467-024-46015-2 38424037 PMC10904840

[5] Stragier H Vandersmissen H Ordies S Thiessen S Mesotten D Peuskens D . Pathophysiological mechanisms underlying early brain injury and delayed cerebral ischemia in the aftermath of aneurysmal subarachnoid hemorrhage: a comprehensive analysis. Front Neurol. (2025) 16:1587091. doi: 10.3389/fneur.2025.1587091 40488203 PMC12142988

[6] Alsbrook DL Di Napoli M Bhatia K Desai M Hinduja A Rubinos CA . Pathophysiology of early brain injury and its association with delayed cerebral ischemia in aneurysmal subarachnoid hemorrhage: a review of current literature. J Clin Med. (2023) 12:1015. doi: 10.3390/jcm12031015 36769660 PMC9918117

[7] Dodd WS Laurent D Dumont AS Hasan DM Jabbour PM Starke RM . Pathophysiology of delayed cerebral ischemia after subarachnoid hemorrhage: a review. J Am Heart Assoc. (2021) 10:e021845. doi: 10.1161/JAHA.121.021845 34325514 PMC8475656

[8] Giovampaola MD Vieno A Higuet A Cavalli I Stella C Coppalini G . Association between systemic inflammation biomarkers and outcomes in patients with aneurysmal subarachnoid hemorrhage. Front Neurol. (2026) 17:1749871. doi: 10.3389/fneur.2026.1749871 41923872 PMC13035489

[9] Song Z Lin F Chen Y Li T Li R Lu J . Inflammatory burden index: association between novel systemic inflammatory biomarkers and prognosis as well as in-hospital complications of patients with aneurysmal subarachnoid hemorrhage. J Inflamm Res. (2023) 16:3911–21. doi: 10.2147/JIR.S416295 37692059 PMC10488670

[10] Yun S Yi HJ Lee DH Sung JH. Systemic inflammation response index and systemic immune-inflammation index for predicting the prognosis of patients with aneurysmal subarachnoid hemorrhage. J Stroke Cerebrovasc Dis. (2021) 30:105861. doi: 10.1016/j.jstrokecerebrovasdis.2021.105861 34034125

[11] Geraghty JR Lung TJ Hirsch Y Katz EA Cheng T Saini NS . Systemic immune-inflammation index predicts delayed cerebral vasospasm after aneurysmal subarachnoid hemorrhage. Neurosurgery. (2021) 89:1071–9. doi: 10.1093/neuros/nyab354 PMC8600162 34560777

[12] Yi HJ Shin DS Kim BT. Dynamic changes of systemic inflammation response index and systemic immune-inflammation index are associated with delayed cerebral ischemia after aneurysmal subarachnoid hemorrhage. J Stroke Cerebrovasc Dis. (2024) 33:107626. doi: 10.1016/j.jstrokecerebrovasdis.2024.107626 38325674

[13] Juvela S Siironen J. D-dimer as an independent predictor for poor outcome after aneurysmal subarachnoid hemorrhage. Stroke. (2006) 37:1451–6. doi: 10.1161/01.STR.0000221710.55467.33 16690901

[14] Boluijt J Meijers JC Rinkel GJ Vergouwen MD. Hemostasis and fibrinolysis in delayed cerebral ischemia after aneurysmal subarachnoid hemorrhage: a systematic review. J Cereb Blood Flow Metab. (2015) 35:724–33. doi: 10.1038/jcbfm.2015.13 25690473 PMC4420861

[15] Fan MC Li HT Sun J Guan D Yang ZJ Feng YG. Preoperative prognostic nutrition index can independently predict the 6-month prognosis of elderly patients undergoing neurosurgical clipping for aneurysmal subarachnoid hemorrhage. Neurosurg Rev. (2023) 46:117. doi: 10.1007/s10143-023-02021-4 37165260

[16] Gel MS Daltaban IS Taser Kanat N Kanat A. First report of validation of the prognostic nutritional index in patients with subarachnoid hemorrhage undergoing endovascular occlusion or surgical clipping. Int J Neurosci. (2025) 135:892–7. doi: 10.1080/00207454.2024.2341908 38626294

[17] Wang X Tu W Li X Wu G. Development and validation of interpretable machine learning models for predicting long-term functional outcomes in elderly patients with aneurysmal subarachnoid hemorrhage. J Clin Neurosci. (2026) 147:111899. doi: 10.1016/j.jocn.2026.111899 41653638

[18] Young RC Biggs JT Ziegler VE Meyer DA A rating scale for mania: reliability validity and sensitivity. Br J Psychiatry. (1978) 133:429–35. doi: 10.1192/bjp.133.5.429 728692

[19] Couret D Boussen S Cardoso D Alonzo A Madec S Reyre A . Comparison of scales for the evaluation of aneurysmal subarachnoid hemorrhage: a retrospective cohort study. Eur Radiol. (2024) 34:7526–36. doi: 10.1007/s00330-024-10814-4 PMC11519170 38836940

[20] Lenkeit A Oppong MD Dinger TF Gümüs M Rauschenbach L Chihi M . Risk factors for poor outcome after aneurysmal subarachnoid hemorrhage in patients with initial favorable neurological status. Acta Neurochir. (2024) 166:93. doi: 10.1007/s00701-024-05968-5 38376665 PMC10879324

[21] Hou Y Fan J Yuan H Zheng H Yang H Li H . Prognostic capacity of the systemic inflammation response index for functional outcome in patients with aneurysmal subarachnoid hemorrhage. Front Neurol. (2023) 14:1054315. doi: 10.3389/fneur.2023.1054315 36937535 PMC10017774

[22] Wang L Chen L Jin Y Cao X Xue L Cheng Q. Clinical value of the low-grade inflammation score in aneurysmal subarachnoid hemorrhage. BMC Neurol. (2023) 23:436. doi: 10.1186/s12883-023-03490-2 PMC10712030 38082254

[23] Raatikainen E Kiiski H Kuitunen A Junttila E Huhtala H Kallonen A . Increased blood coagulation is associated with poor neurological outcome in aneurysmal subarachnoid hemorrhage. J Neurol Sci. (2024) 458:122943. doi: 10.1016/j.jns.2024.122943 38422781

[24] Guo Q Chen H Lin S Gong Z Song Z Chen F. Innovative prognostication: a novel nomogram for post-interventional aneurysmal subarachnoid hemorrhage patients. Front Neurol. (2024) 15:1410735. doi: 10.3389/fneur.2024.1410735 39228509 PMC11369945

[25] Hu P Xu Y Liu Y Li Y Ye L Zhang S . An externally validated dynamic nomogram for predicting unfavorable prognosis in patients with aneurysmal subarachnoid hemorrhage. Front Neurol. (2021) 12:683051. doi: 10.3389/fneur.2021.683051 34512505 PMC8426570

[26] Zhou W Liao X Shi H Wang M Xiao Y Yu X . Prediction of the 180 day functional outcomes in aneurysmal subarachnoid hemorrhage using an optimized XGBoost model. Sci Rep. (2025) 15:20833. doi: 10.1038/s41598-025-05432-z 40594335 PMC12217176

[27] de Winkel J Maas CCHM Roozenbeek B van Klaveren D Lingsma HF. Pitfalls of single-study external validation illustrated with a model predicting functional outcome after aneurysmal subarachnoid hemorrhage. BMC Med Res Methodol. (2024) 24:176. doi: 10.1186/s12874-024-02280-9 39118007 PMC11308226

