# Supplementary material for: Inflammation, coagulation, and nutritional reserves refine 90-day prognostic stratification after ruptured intracranial aneurysm repair
Source: Front Nutr. 2026 Sep 16;13:1918951. doi: 10.3389/fnut.2026.1918951 (PMC13623685; doi:10.3389/fnut.2026.1918951)
Supplement: Supplementary file 1 [file Supplementary_file_1.docx]

**Supplementary Table S1. Variance inflation factor (VIF) assessment for candidate predictors included in multivariable models**

| **Predictor variable** | **VIF** | **Tolerance** | **Model inclusion** |
| --- | --- | --- | --- |
| Hunt–Hess grade IV–V | 1.62 | 0.617 | Included |
| Modified Fisher grade 3–4 | 1.48 | 0.676 | Included |
| Acute hydrocephalus | 1.35 | 0.741 | Included |
| SII (per 500-unit increase) | 1.84 | 0.543 | Included |
| D-dimer (per 1 mg/L increase) | 1.61 | 0.621 | Included |
| PNI (per 5-point increase) | 1.45 | 0.69 | Included |
| DCI (Updated In-Hospital Model only) | 1.52 | 0.658 | Included |
| Treatment modality (coiling vs. clipping) | 1.18 | 0.847 | Included |
| SIRI (per 1-unit increase) | **6.82** | **0.147** | Excluded due to collinearity with SII |

Supplementary Table S2. Baseline characteristics of patients included and excluded due to loss to 90-day follow-up

| **Variable** | **Included (n=209)** | **Lost follow-up (n=13)** | **P value** |
| --- | --- | --- | --- |
| Age, years | 58.6±11.8 | 61.2±12.6 | 0.41 |
| Male sex | 92 (44.0%) | 7 (53.8%) | 0.49 |
| Hunt–Hess grade IV–V | 65 (31.1%) | 5 (38.5%) | 0.57 |
| Modified Fisher grade 3–4 | 128 (61.2%) | 9 (69.2%) | 0.56 |
| Acute hydrocephalus | 61 (29.2%) | 5 (38.5%) | 0.47 |
| DCI | 45 (21.5%) | 4 (30.8%) | 0.46 |
| SII | 1380 (860–2120) | 1560 (990–2440) | 0.62 |
| D-dimer (mg/L) | 1.85 (0.98–3.82) | 2.10 (1.10–4.05) | 0.71 |
| PNI | 46.3±5.4 | 44.9±5.8 | 0.35 |

**Supplementary Table S3. Baseline candidate variables with missing data and imputation strategy (N=209)**

| Candidate Variable | Available Cases, n (%) | Missing Cases, n (%) | Missing Proportion (%) | Primary Imputation / Management Strategy |
| --- | --- | --- | --- | --- |
| Inflammatory & Infection Biomarkers |  |  |  |  |
| C-Reactive Protein (CRP) | 206 (98.6) | 3 (1.4) | 1.40% | Complete-case analysis (<5%) |
| Procalcitonin (PCT) | 178 (85.2) | 31 (14.8) | 14.80% | MICE (chained equations, m=20) |
| White Blood Cell Count (WBC) | 205 (98.1) | 4 (1.9) | 1.90% | Complete-case analysis (<5%) |
| Absolute Monocyte Count | 202 (96.7) | 7 (3.3) | 3.30% | Complete-case analysis (<5%) |
| Coagulation Parameters |  |  |  |  |
| D-dimer | 196 (93.8) | 13 (6.2) | 6.20% | MICE (chained equations, m=20) |
| Fibrinogen | 198 (94.7) | 11 (5.3) | 5.30% | MICE (chained equations, m=20) |
| Activated Partial Thromboplastin Time (APTT) | 203 (97.1) | 6 (2.9) | 2.90% | Complete-case analysis (<5%) |
| Nutritional & Liver Parameters |  |  |  |  |
| Serum Albumin | 201 (96.2) | 8 (3.8) | 3.80% | Complete-case analysis (<5%) |
| Total Protein | 200 (95.7) | 9 (4.3) | 4.30% | Complete-case analysis (<5%) |
| Radiological & Clinical Features |  |  |  |  |
| Aneurysm Maximum Diameter | 194 (92.8) | 15 (7.2) | 7.20% | MICE (chained equations, m=20) |
| Aneurysm Neck Width | 191 (91.4) | 18 (8.6) | 8.60% | MICE (chained equations, m=20) |
| Time from Onset to Treatment | 197 (94.3) | 12 (5.7) | 5.70% | MICE (chained equations, m=20) |

Abbreviations: MICE, Multiple Imputation by Chained Equations; PCT, procalcitonin; CRP, C-reactive protein.

Table Footnotes:
Missing Data Handling: Variables with <5% missing values were analyzed using complete-case analysis. Variables with 5%–20% missingness were imputed using MICE with 20 imputed datasets.

Imputation Model Specification: The MICE model included all primary predictors (Hunt–Hess grade, modified Fisher grade, acute hydrocephalus, SII, D-dimer, PNI, and DCI), auxiliary baseline covariates, and the primary outcome variable (90-day mRS). Regression estimates from the imputed datasets were combined using Rubin’s rules.

Exclusion Criterion: No primary candidate variable had >20% missing data; therefore, no variables required exclusion based on the predefined threshold.

**Supplementary Table S4. Baseline laboratory biological parameters and composite indices according to 90-day neurological recovery**

| Laboratory Biomarker | Total Cohort (N=209) | Favorable Recovery (n=143) | Unfavorable Recovery (n=66) | P Value | Standardized Mean Difference (SMD) |
| --- | --- | --- | --- | --- | --- |
| Systemic Biomarkers |  |  |  |  |  |
| PNI | 46.3±5.4 | 48.6±4.2 | 41.2±4.8 | <0.001 | -1.02 |
| SII (×109/L) | 1380 (860–2120) | 1120 (760–1540) | 2150 (1480–2980) | <0.001 | 0.91 |
| D-dimer (mg/L) | 1.85 (0.98–3.82) | 1.45 (0.82–2.60) | 3.82 (2.10–6.45) | <0.001 | 0.83 |
| Albumin (g/L) | 39.8±4.2 | 41.2±3.8 | 36.8±4.1 | <0.001 | -0.78 |
| SIRI (×109/L) | 2.15 (1.32–3.68) | 1.82 (1.15–2.95) | 3.25 (2.10–5.12) | <0.001 | 0.72 |
| CAR | 0.28 (0.12–0.65) | 0.21 (0.09–0.48) | 0.52 (0.24–1.18) | <0.001 | 0.69 |
| CRP (mg/L) | 11.2 (4.5–28.6) | 8.6 (3.8–20.2) | 21.5 (9.2–45.8) | <0.001 | 0.63 |
| NLR | 5.8 (3.6–9.2) | 4.9 (3.2–7.5) | 8.6 (5.2–13.8) | <0.001 | 0.58 |
| Fibrinogen (g/L) | 3.85±0.92 | 3.68±0.85 | 4.22±0.98 | <0.001 | 0.52 |
| PLR | 148 (105–210) | 138 (98–188) | 182 (125–258) | <0.001 | 0.45 |
| PCT (ng/mL) | 0.12 (0.05–0.32) | 0.09 (0.04–0.22) | 0.21 (0.08–0.58) | 0.002 | 0.39 |
| PT (s) | 11.8±1.1 | 11.7±1.0 | 11.9±1.2 | 0.218 | 0.12 |

Continuous variables are expressed as mean ± SD or median (interquartile range, IQR).

Supplementary Table S5. Distribution of treatment modality according to aneurysm characteristics

| **Aneurysm characteristic** | **Clipping (n=94)** | **Coiling (n=115)** | **P value** |
| --- | --- | --- | --- |
| Anterior circulation aneurysm | 78 (46.7%) | 89 (53.3%) | 0.62 |
| Posterior circulation aneurysm | 16 (38.1%) | 26 (61.9%) | 0.41 |
| Wide-neck aneurysm | 27 (40.9%) | 39 (59.1%) | 0.48 |
| Irregular morphology | 40 (46.5%) | 46 (53.5%) | 0.87 |
| Maximum diameter ≥10 mm | 43 (45.7%) | 51 (44.3%) | 0.84 |

**Supplementary Table S6. Sensitivity analysis retaining onset-to-treatment time in the multivariable model**

| **Variable** | **Multivariable OR (95% CI)** | **P value** |
| --- | --- | --- |
| Hunt–Hess grade IV–V | 3.74 (1.79–7.82) | <0.001 |
| Modified Fisher grade 3–4 | 2.69 (1.25–5.82) | 0.011 |
| Acute hydrocephalus | 2.55 (1.21–5.38) | 0.014 |
| SII (per 500-unit increase) | 1.31 (1.13–1.52) | <0.001 |
| D-dimer (per 1 mg/L increase) | 1.21 (1.06–1.38) | 0.004 |
| PNI (per 5-point increase) | 0.69 (0.56–0.85) | <0.001 |
| Treatment modality (coiling vs. clipping) | 1.11 (0.60–2.05) | 0.741 |
| Time from onset to treatment (per 12 h increase) | 1.15 (0.97–1.37) | 0.104 |

**Supplementary Table S7. Sensitivity analysis of biomarker associations stratified by time from ictus to first blood sampling**

| **Biomarker** | **≤6 h aOR (95% CI)** | **P value** | **6–24 h aOR (95% CI)** | **P value** | **P for interaction** |
| --- | --- | --- | --- | --- | --- |
| SII (per 500-unit increase) | 1.31 (1.10–1.56) | 0.003 | 1.27 (1.09–1.48) | 0.002 | 0.72 |
| D-dimer (per 1 mg/L increase) | 1.20 (1.02–1.42) | 0.028 | 1.18 (1.03–1.35) | 0.016 | 0.81 |
| PNI (per 5-point increase) | 0.69 (0.53–0.89) | 0.006 | 0.72 (0.58–0.90) | 0.004 | 0.68 |

A worst-case sensitivity analysis was performed by assigning all patients lost to follow-up as having unfavorable neurological outcomes (mRS 3–6). Under this conservative assumption, the associations between admission biomarkers and poor neurological recovery remained consistent with the primary analysis. The adjusted effect estimates were minimally changed for SII (aOR=1.29, 95% CI: 1.12–1.49, P<0.001), D-dimer (aOR=1.20, 95% CI: 1.06–1.37, P=0.004), and PNI (aOR=0.70, 95% CI: 0.57–0.86, P<0.001), supporting the robustness of the findings against potential outcome misclassification caused by missing follow-up.(Supplementary Table S8)

Supplementary Table S8. Sensitivity analysis using worst-case imputation for missing follow-up outcomes

| **Biomarker** | **Primary analysis aOR (95% CI)** | **Worst-case analysis aOR (95% CI)** |
| --- | --- | --- |
| SII (per 500-unit increase) | 1.32 (1.14–1.53) | 1.29 (1.12–1.49) |
| D-dimer (per 1 mg/L increase) | 1.22 (1.07–1.39) | 1.20 (1.06–1.37) |
| PNI (per 5-point increase) | 0.68 (0.55–0.84) | 0.70 (0.57–0.86) |

**Supplementary Figure S1. Nonlinear dose–response association and diagnostic performance of admission prognostic nutritional index (PNI) for unfavorable neurological recovery.**

(A) Restricted cubic spline analysis illustrating the nonlinear association between admission PNI and the risk of unfavorable neurological recovery (modified Rankin Scale [mRS] 3–6 at 90 days). The solid blue line represents the adjusted odds ratio (OR), and the shaded area indicates the corresponding 95% confidence interval (CI). The reference value was set at PNI = 45 (OR = 1). A significant overall association and nonlinear relationship were observed between PNI and poor neurological recovery (P_overall < 0.001; P_non-linear = 0.028). The risk of unfavorable recovery increased progressively when PNI decreased below approximately 45 points.

(B) Receiver operating characteristic (ROC) curve analysis evaluating the predictive performance of admission PNI for unfavorable neurological recovery. The optimal PNI cutoff identified by the Youden index was 45.2, yielding a sensitivity of 80.3% and specificity of 76.2% (AUC = 0.84, 95% CI: 0.78–0.90).

Abbreviations: PNI, prognostic nutritional index; mRS, modified Rankin Scale; ROC, receiver operating characteristic; AUC, area under the curve; OR, odds ratio; CI, confidence interval.

**Supplementary Figure S2. Association between admission prognostic nutritional index (PNI) and in-hospital nutritional trajectory.**

(A) Comparison of serum albumin decline during hospitalization between patients with higher and lower admission PNI. Patients were stratified according to the previously identified PNI cutoff value of 45.2. Patients with PNI <45.2 exhibited a greater decrease in serum albumin levels from admission to discharge compared with those with PNI ≥45.2 (−6.8 ± 3.1 g/L vs. −3.2 ± 2.5 g/L, P<0.001), indicating increased nutritional depletion during the acute hospitalization period.

(B) Comparison of enteral feeding duration according to admission PNI category. Patients with lower admission PNI (<45.2) required longer enteral nutritional support than those with PNI ≥45.2 (median 12 [IQR: 8–18] days vs. 8 [IQR: 5–12] days, P<0.001), suggesting greater nutritional support requirements among patients with reduced baseline nutritional reserve.

Abbreviations: PNI, prognostic nutritional index; IQR, interquartile range. ΔAlbumin, change in serum albumin concentration from admission to discharge.
